# Rare coding variants and X-linked loci associated with age at menarche

**DOI:** 10.1038/ncomms8756

**Published:** 2015-08-04

**Authors:** Kathryn L. Lunetta, Felix R. Day, Patrick Sulem, Katherine S. Ruth, Joyce Y. Tung, David A. Hinds, Tõnu Esko, Cathy E. Elks, Elisabeth Altmaier, Chunyan He, Jennifer E. Huffman, Evelin Mihailov, Eleonora Porcu, Antonietta Robino, Lynda M. Rose, Ursula M. Schick, Lisette Stolk, Alexander Teumer, Deborah J. Thompson, Michela Traglia, Carol A. Wang, Laura M. Yerges-Armstrong, Antonis C. Antoniou, Caterina Barbieri, Andrea D. Coviello, Francesco Cucca, Ellen W. Demerath, Alison M. Dunning, Ilaria Gandin, Megan L. Grove, Daniel F. Gudbjartsson, Lynne J. Hocking, Albert Hofman, Jinyan Huang, Rebecca D. Jackson, David Karasik, Jennifer Kriebel, Ethan M. Lange, Leslie A. Lange, Claudia Langenberg, Xin Li, Jian'an Luan, Reedik Mägi, Alanna C. Morrison, Sandosh Padmanabhan, Ailith Pirie, Ozren Polasek, David Porteous, Alex P. Reiner, Fernando Rivadeneira, Igor Rudan, Cinzia F. Sala, David Schlessinger, Robert A. Scott, Doris Stöckl, Jenny A. Visser, Uwe Völker, Diego Vozzi, James G. Wilson, Marek Zygmunt, EPIC-InterAct Consortium, Nita G. Forouhi, Nicola D. Kerrison, Stephen J. Sharp, Matt Sims, Inês Barroso, Panos Deloukas, Mark I. McCarthy, Larraitz Arriola, Beverley Balkau, Aurelio Barricarte, Heiner Boeing, Paul W. Franks, Carlos Gonzalez, Sara Grioni, Rudolf Kaaks, Timothy J. Key, Carmen Navarro, Peter M. Nilsson, Kim Overvad, Domenico Palli, Salvatore Panico, J Ramón Quirós, Olov Rolandsson, Carlotta Sacerdote, María‐José Sánchez, Nadia Slimani, Anne Tjonneland, Rosario Tumino, Daphne L. van der A, Yvonne T. van der Schouw, Elio Riboli, Generation Scotland, Blair H. Smith, Archie Campbell, Ian J. Deary, Andrew M. McIntosh, Eric Boerwinkle, Julie E. Buring, Laura Crisponi, Douglas F. Easton, Caroline Hayward, Frank B. Hu, Simin Liu, Andres Metspalu, Craig E. Pennell, Paul M. Ridker, Konstantin Strauch, Elizabeth A. Streeten, Daniela Toniolo, André G. Uitterlinden, Sheila Ulivi, Henry Völzke, Nicholas J. Wareham, Melissa Wellons, Nora Franceschini, Daniel I. Chasman, Unnur Thorsteinsdottir, Anna Murray, Kari Stefansson, Joanne M. Murabito, Ken K. Ong, John R. B. Perry

**Affiliations:** 1 Boston University School of Public Health, Department of Biostatistics, Boston, Massachusetts 02118, USA; 2 NHLBI's and Boston University's Framingham Heart Study, Framingham, Massachusetts 01702-5827, USA; 3 MRC Epidemiology Unit, University of Cambridge School of Clinical Medicine, Box 285 Institute of Metabolic Science, Cambridge Biomedical Campus, Cambridge CB2 0QQ, UK; 4 deCODE genetics/Amgen, Inc., Reykjavik IS-101, Iceland; 5 Genetics of Complex Traits, University of Exeter Medical School, University of Exeter, Exeter EX1 2LU, UK; 6 23andMe Inc., 1390 Shorebird Way, Mountain View, California 94043, USA; 7 Estonian Genome Center, University of Tartu, Tartu 51010, Estonia; 8 Division of Endocrinology, Boston Children's Hospital, Boston, MA 02115, USA; 9 Department of Genetics, Harvard Medical School, Boston, MA 02115, USA; 10 Broad Institute of the Massachusetts Institute of Technology and Harvard University, 140, Cambridge, MA 02142, USA; 11 Research Unit of Molecular Epidemiology, Helmholtz Zentrum München–German Research Center for Environmental Health, Neuherberg 85764, Germany; 12 Institute of Genetic Epidemiology, Helmholtz Zentrum München–German Research Center for Environmental Health, Neuherberg 85764, Germany; 13 Department of Epidemiology, Indiana University Richard M. Fairbanks School of Public Health, Indianapolis, IN 46202, USA; 14 Indiana University Melvin and Bren Simon Cancer Center, Indianapolis, IN 46202, USA; 15 Medical Research Council Human Genetics Unit, Institute of Genetics and Molecular Medicine, University of Edinburgh, Edinburgh EH4 2XU, UK; 16 Institute of Genetics and Biomedical Research, National Research Council, Cagliari, Sardinia 09042, Italy; 17 University of Sassari, Department of Biomedical Sciences, Sassari, Sassari 07100, Italy; 18 Center for Statistical Genetics, Ann Arbor, University of Michigan, Michigan 48109-2029, USA; 19 Institute for Maternal and Child Health—IRCCS “Burlo Garofolo”, Trieste 34137, Italy; 20 Division of Preventive Medicine, Brigham and Women's Hospital, Boston, MA 02215; 21 Fred Hutchinson Cancer Research Center, Public Health Sciences Division, Seattle, WA 98109-1024, USA; 22 Department of Internal Medicine, Erasmus MC, Rotterdam 3015GE, the Netherlands; 23 Institute for Community Medicine, University Medicine Greifswald, Greifswald 17475, Germany; 24 Centre for Cancer Genetic Epidemiology, Department of Public Health and Primary Care, University of Cambridge, CB1 8RN, UK; 25 Division of Genetics and Cell Biology, San Raffaele Scientific Institute, Milano 20132, Italy; 26 School of Women's and Infants' Health, The University of Western Australia, WA-6009, Australia; 27 Program in Personalized Medicine, Division of Endocrinology, Diabetes and Nutrition—University of Maryland School of Medicine, Baltimore, MD 21201, USA; 28 Boston University School of Medicine, Department of Medicine, Sections of Preventive Medicine and Endocrinology, Boston, MA, USA; 29 Division of Epidemiology & Community Health, University of Minnesotta, Minneapolis, MN 55455, USA; 30 Centre for Cancer Genetic Epidemiology, Department of Oncology, University of Cambridge, Cambridge CB1 8RN, UK; 31 Department of Clinical Medical Sciences, Surgical and Health, University of Trieste, Trieste 34149, Italy; 32 Human Genetics Center, School of Public Health, The University of Texas Health Science Center at Houston, Houston, TX 77030, USA; 33 School of Engineering and Natural Sciences, University of Iceland, Reykjavik IS-101, Iceland; 34 Musculoskeletal Research Programme, Division of Applied Medicine, University of Aberdeen, Aberdeen AB25 2ZD, UK; 35 Genetic Epidemiology Unit Department of Epidemiology, Erasmus MC, Rotterdam 3015 GE, the Netherlands; 36 State Key Laboratory of Medical Genomics, Shanghai Institute of Hematology, Rui Jin Hospital, Shanghai Jiao Tong University School of Medicine, Shanghai 200025, China; 37 Department of Internal Medicine, The Ohio State University, Columbus, Ohio 43210, USA; 38 Hebrew SeniorLife Institute for Aging Research, Boston, MA 02131, USA; 39 Harvard Medical School, Boston, MA 02115, USA; 40 German Center for Diabetes Research, Neuherberg 85764, Germany; 41 Department of Genetics, University of North Carolina, Chapel Hill, NC 27599, USA; 42 Department of Biostatistics, University of North Carolina, Chapel Hill, NC 27599, USA; 43 Department of Epidemiology, Harvard School of Public Health, Boston, MA 02115, USA; 44 British Heart Foundation Glasgow Cardiovascular Research Centre, Institute of Cardiovascular and Medical Sciences, College of Medical, Veterinary and Life Sciences, University of Glasgow, Glasgow G12 8TA, UK; 45 Faculty of Medicine, University of Split, Split, Croatia; 46 Medical Genetics Section, Centre for Genomic and Experimental Medicine, Institute of Genetics and Molecular Medicine, University of Edinburgh, Edinburgh EH4 2XU, UK; 47 Institute for Population Health Sciences and Informatics, University of Edinburgh, Teviot Place, Edinburgh EH8 9AG, Scotland; 48 National Institute on Aging, Intramural Research Program, Baltimore, MD 20892, USA; 49 Institute of Epidemiology II, Helmholtz Zentrum München - German Research Center for Environmental Health, Neuherberg 85764, Germany; 50 Interfaculty Institute for Genetics and Functional Genomics, University Medicine Greifswald, Greifswald 17475, Germany; 51 Department of Physiology and Biophysics, University of Mississippi Medical Center, Jackson, MS 39216, USA; 52 Department of Obstetrics and Gynecology, University Medicine Greifswald, Greifswald 17475, Germany; 53 Channing Division of Network Medicine, Department of Medicine, Brigham and Women’s Hospital and Harvard Medical School, Boston, MA 02115, USA; 54 Department of Nutrition, Harvard School of Public Health, Boston, MA 02115, USA; 55 Departments of Epidemiology and Medicine Brown University, Brown University, Providence, RI 02912, USA; 56 Institute of Molecular and Cell Biology, University of Tartu, Tartu 51010, Estonia; 57 Institute of Medical Informatics, Biometry and Epidemiology, Chair of Genetic Epidemiology, Ludwig-Maximilians-Universität, Munich 81377, Germany; 58 Department of Medicine, Vanderbilt University Medical Center, Nashville, Tennessee 37203, USA; 59 Department of Epidemiology, University of North Carolina, Chapel Hill, NC 27599, USA; 60 Faculty of Medicine, University of Iceland, Reykjavik IS-101, Iceland; 61 Boston University School of Medicine, Department of Medicine, Section of General Internal Medicine, Boston, MA 02118, USA; 62 Department of Paediatrics, University of Cambridge, Cambridge CB2 0QQ, UK; 63 The Wellcome Trust Sanger Institute, Cambridge CB10 1SA, UK; 64 University of Cambridge Metabolic Research Laboratories, Cambridge CB2 0QQ, UK; 65 Oxford Centre for Diabetes, Endocrinology and Metabolism (OCDEM), University of Oxford, OX3 7LJ, UK; 66 Wellcome Trust Centre for Human Genetics, University of Oxford, Oxford OX3 7BN, UK; 67 Oxford NIHR Biomedical Research Centre, Oxford OX3 7LJ, UK; 68 Public Health Division of Gipuzkoa, San Sebastian 20013, Spain; 69 Instituto BIO‐Donostia, Basque Government, Donostia, San Sebastian 20014, Spain; 70 CIBER Epidemiología y Salud Pública (CIBERESP), Madrid 28029, Spain; 71 Inserm, CESP, U1018, Villejuif, cedex 94807, France; 72 Univ Paris‐Sud, UMRS 1018, Villejuif F-94805, France; 73 Navarre Public Health Institute (ISPN), Pamplona 31003, Spain; 74 German Institute of Human Nutrition Potsdam‐Rehbruecke, Nuthetal 14558, Germany; 75 Department of Clinical Sciences, Lund University, Malmö S-205 02, Sweden; 76 Department of Public Health and Clinical Medicine, Umeå University, Umeå 90187, Sweden; 77 Catalan Institute of Oncology (ICO), Badalona, Barcelona 08916, Spain; 78 Epidemiology and Prevention Unit, Milan 20133, Italy; 79 German Cancer Research Centre (DKFZ), Heidelberg 69120, Germany; 80 Cancer Epidemiology Unit, Nuffield Department of Population Health, University of Oxford, OX3 7LF, UK; 81 Department of Epidemiology, Murcia Regional Health Council, Murcia 30008, Spain; 82 Unit of Preventive Medicine and Public Health, School of Medicine, University of Murcia, Espinardo 30100, Spain; 83 Department of Public Health, Section for Epidemiology, Aarhus University, Aarhus C DK-8000, Denmark; 84 Aalborg University Hospital, Aalborg 9100, Denmark; 85 Cancer Research and Prevention Institute (ISPO), Florence 50141, Italy; 86 Dipartimento di Medicina Clinica e Chirurgia, Federico II University, Naples 80131, Italy; 87 Public Health Directorate, Oviedo-Asturias 33006, Spain; 88 Unit of Cancer Epidemiology, Citta' della Salute e della Scienza Hospital‐University of Turin and Center for Cancer Prevention (CPO), Torino 10126, Italy; 89 Human Genetics Foundation (HuGeF), Torino 10126, Italy; 90 Andalusian School of Public Health, Granada 18080, Spain; 91 Instituto de Investigación Biosanitaria de Granada (Granada.ibs), Granada 18012, Spain; 92 International Agency for Research on Cancer, Lyon, Cedex 08 69372, France; 93 Danish Cancer Society Research Center, Copenhagen 2100, Denmark; 94 ASP, Ragusa 97100, Italy; 95 Ragusa Cancer Registry, Aire Onlus, Ragusa 97100, Italy; 96 National Institute for Public Health and the Environment (RIVM), Bilthoven 3720 BA, The Netherlands; 97 University Medical Center Utrecht, Utrecht 3508 GA, the Netherlands; 98 School of Public Health, Imperial College London, W2 1PG, UK; 99 Centre for Genomic and Experimental Medicine, Institute of Genetics and Molecular Medicine, University of Edinburgh, Edinburgh EH4 2XU, UK; 100 Centre for Cognitive Ageing and Cognitive Epidemiology, University of Edinburgh, Edinburgh, UK; 101 Department of Psychology, University of Edinburgh, EH8 9JZ, UK; 102 Division of Psychiatry, University of Edinburgh, Edinburgh, EH10 5HF, UK

## Abstract

More than 100 loci have been identified for age at menarche by genome-wide association studies; however, collectively these explain only ∼3% of the trait variance. Here we test two overlooked sources of variation in 192,974 European ancestry women: low-frequency protein-coding variants and X-chromosome variants. Five missense/nonsense variants (in *ALMS1*/*LAMB2*/*TNRC6A/TACR3/PRKAG1*) are associated with age at menarche (minor allele frequencies 0.08–4.6%; effect sizes 0.08–1.25 years per allele; *P*<5 × 10^−8^). In addition, we identify common X-chromosome loci at *IGSF1* (rs762080, *P*=9.4 × 10^−13^) and *FAAH2* (rs5914101, *P*=4.9 × 10^−10^). Highlighted genes implicate cellular energy homeostasis, post-transcriptional gene silencing and fatty-acid amide signalling. A frequently reported mutation in *TACR3* for idiopathic hypogonatrophic hypogonadism (p.W275X) is associated with 1.25-year-later menarche (*P*=2.8 × 10^−11^), illustrating the utility of population studies to estimate the penetrance of reportedly pathogenic mutations. Collectively, these novel variants explain ∼0.5% variance, indicating that these overlooked sources of variation do not substantially explain the ‘missing heritability’ of this complex trait.

## Abstract (web summary)

Previous studies have linked over 100 genomic loci to age-at-menarche but that work was restricted to common autosomal variation. Here, Lunetta *et al*. identify associations with rare protein-coding and X-linked variants, implicating new mechanisms that regulate puberty timing.

## Graphical abstract

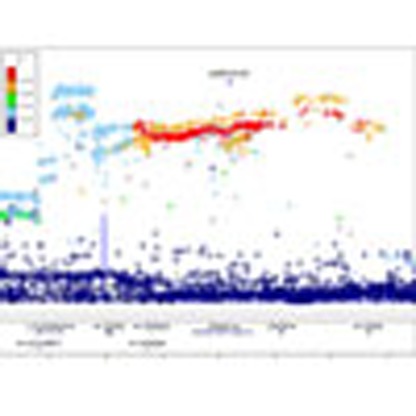

Age at menarche, the onset of first menstruation in females, indicates the start of reproductive maturity and is a commonly reported marker of pubertal timing. One hundred and six genomic loci for this highly heritable trait have been mapped by genome-wide association studies (GWAS), implicating many previously unsuspected mechanisms^1^. However, to date that approach has been limited to consideration of only those genetic variants captured by autosomal HapMap2 reference panels. In particular, like most GWAS for other complex traits, previous GWAS for age at menarche provided poor coverage for low-frequency variants and omitted sex chromosome data.

Here we report a dual strategy for assessing genetic variation overlooked by those prior efforts: low-frequency protein-coding variants genotyped by large-scale exome-focussed arrays and high-density X-chromosome single-nucleotide polymorphism (SNP) genotyping and imputation. We identify several new associations between rare protein-coding and X-linked variants with age at menarche in women of European ancestry. The findings implicate new mechanisms that regulate puberty timing, but collectively these novel variants explained only ∼0.5% of the variance, indicating that these often overlooked sources of variation that do not substantially explain the ‘missing heritability’ of this complex trait.

## Results

**Figure 1:**
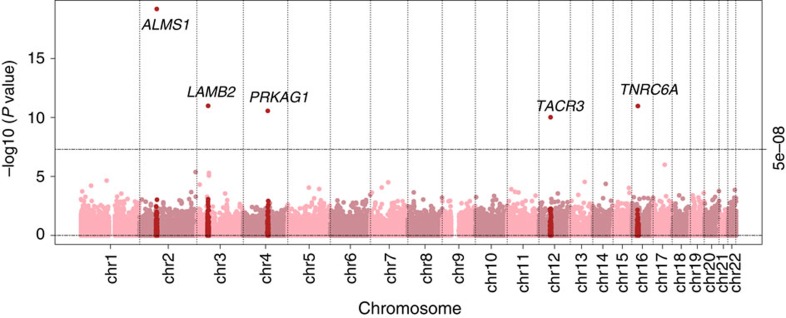
A ‘Manhattan plot’ of menarche association statistics for the genotyped low-frequency exome array variants. Test statistics are shown from the exome-chip discovery-phase samples, with the exception of the five labelled loci that indicate results from the combined discovery and replication set.

From the exome array studies, 61,734 low-frequency (minor allele frequency (MAF) <5%) variants passed quality-control (QC) criteria in a combined sample of up to 76,657 women of European ancestry from 19 studies (Supplementary Table 1). Gene-based burden and SKAT tests that aggregate the effects of variants with MAF<1% yielded no significant associations with age at menarche. A linear regression test was used to derive all *P* values obtained in this study. Meta-analysis of individual variant associations with questionnaire-reported variation in age at menarche (restricted to the ages of 9–17 years) in this discovery phase identified one signal at genome-wide statistical significance (*P*<5 × 10^−8^); this was a rare missense variant in the Alström’s syndrome gene (*ALMS1*, rs45501594, p.T3544S, MAF 1%; *P*=4.6 × 10^−10^). For follow-up testing in up to 116,317 independent women of European ancestry from the deCODE (Diabetes Epidemiology: Collaborative analysis of Diagnostic criteria in Europe) and 23andMe studies, we selected rs45501594 and 23 other variants that met the following criteria: protein coding, present in over half of the exome array studies, and with association *P*<5 × 10^−4^. In the follow-up samples, 7 of the 20 variants that passed QC showed directionally concordant confirmatory associations with *P*<0.05, of which five reached genome-wide significance in a combined meta-analysis of discovery phase and follow-up data (Table 1, Fig. 1, Supplementary Fig. 1). No significant heterogeneity between studies was observed at any of these loci (Supplementary Fig. 2).

The rare missense variant in *ALMS1* (rs45501594, Supplementary Fig. 3) remained the strongest signal identified using exome array studies (combined: *P*=6.8 × 10^−20^). In the follow-up samples, each rare allele was associated with 0.23-year-later age at menarche, an effect size more than double that of any genetic variant previously reported for puberty timing in the general population. This strong signal was not detected by the previous HapMap2-based GWAS as it is poorly tagged by common SNPs in that reference panel (maximum proxy SNP, *r*^2^=0.24). Deleterious mutations in this gene cause Alström’s syndrome (OMIM no. 203800), a rare, autosomal-recessive disorder characterized by cone–rod dystrophy, sensorineural hearing loss, dilated cardiomyopathy, childhood obesity, insulin resistance, diabetes mellitus, hypogonadotropic hypogonadism in males, menstrual irregularities and early puberty in females, and short stature in adulthood^2^. Hypogonadism was also invariably observed in an *ALMS1* gene-trapped mouse model^3^.

The variant with largest effect was a rare stop-gain mutation in the tachykinin receptor 3 gene (*TACR3*; rs144292455, MAF=0.08%, combined *P*=2.8 × 10^−11^, Supplementary Fig. 3); in follow-up samples each rare allele was associated with 1.25-year-later age at menarche. Common HapMap2 SNPs at the *TACR3* locus were previously associated with age at menarche^1^; however, the rare variant rs144292455 is not tagged by the HapMap2 or conventional 1000G imputation (it was directly genotyped in 23andMe and was imputed in deCODE). Statistical independence was confirmed by observing significant association with the common *TACR3* SNP in a sensitivity analysis within a participating study (Women's Genome Health Study (WGHS), Supplementary Table 1) that excluded rare allele carriers. The rare allele causes a premature stop codon (p.W275X) in the fifth transmembrane segment of the 465 amino-acid receptor for the neuropeptide neurokinin B, and is the most frequently reported *TACR3* mutation in the rare reproductive disorder idiopathic hypogonadotropic hypogonadism (idiopathic hypogonadotropic hypogonadism (IHH), OMIM no. 614840)^4^. Both homozygous and heterozygous p.W275X variants have been reported in male IHH cases with features of ‘early androgen deficiency’; however, notably the heterozygous cases showed evidence of spontaneous neuroendocrine recovery. Our findings suggest that heterozygous p.W275X variants contribute to the normal variation in puberty timing, whereas homozygous inheritance or possibly compound heterozygosity is required for IHH.

A low-frequency missense variant in the *LAMB2* gene was associated with 0.08-year-later age at menarche (rs35713889, p.G914R, MAF 4%; *P*=1.1 × 10^−11^; Supplementary Fig. 3). In the same region (3p21.31) we previously reported a HapMap2 GWAS locus for age at menarche (locus 19a and 19b in ref. 1); however, the low-frequency variant rs35713889 is poorly tagged by common HapMap2 SNPs (the best proxy rs1134043, *r*^2^=0.24, was reportedly not associated with age at menarche: *P*=0.35 (ref. 1)). The strongest reported^1^ HapMap2 signal at this locus is only weakly correlated with rs35713889 (rs3870341, MAF=26%; *r*^2^=0.07, distance 422 kb), and both signals remained significant when jointly tested in a follow-up sample of 76,831 women from the 23andMe study (in separate models: rs35713889: *β*=0.08 years per allele, *P*=0.0001 and rs3870341: *β*=0.04, *P*=4.5 × 10^−5^; in the joint model: rs35713889: *β*=0.06, *P*=0.004 and rs3870341: *β*=0.03, *P*=0.001). *LAMB2* encodes one of 15 subunits of Laminin, an extracellular matrix glycoprotein with a key role in the attachment, migration and organization of cells into tissues during embryonic development. Rare recessive mutations in *LAMB2* cause Pierson’s syndrome (OMIM no. 609049), a disorder characterized by congenital nephrotic syndrome and ocular anomalies, typically with microcoria^5^; neurological abnormalities are also described likely because of cortical laminar disorganization^6^. Common variants in/near other Laminin genes have been reported for a broad range of complex traits, including type 2 diabetes^7^, refractive error^8^, colorectal cancer^9^, IgG glycosylation^10^, ulcerative colitis^11^ and coffee consumption^12^.

A low-frequency missense variant in the *TNRC6A* gene was associated with later age at menarche (rs113388806; p.Q1112H; MAF 4.7%; *β*=0.08 years per allele; *P*=1.1 × 10^−11^; Supplementary Fig. 3). This signal was only moderately well tagged by common HapMap2 SNPs (best proxy: rs12447003, *r*^2^=0.36, reported association with age at menarche: *P*=0.0005 (ref. 1)). *TNRC6A* encodes an Argonaute-navigator protein, responsible for post-transcriptional gene silencing through RNA interference and microRNA pathways^13^. This finding further extends the range of epigenetic mechanisms implicated in the regulation of puberty^14^.

A low-frequency missense variant in *PRKAG1* was associated with earlier age at menarche (rs1126930; p.T98S; MAF 3.4%; *β*=−0.09 years per allele, *P*=9.6 × 10^−11^; Supplementary Fig. 3). This low-frequency variant is only moderately well tagged by common HapMap2 SNPs (max *r*^2^=0.36), which reportedly showed subgenome-wide significant association with age at menarche (rs11837234, *P*=3.1 × 10^−6^)^1^. This *PRKAG1* missense variant is in the same locus as, but not correlated to, a reported^1^ common signal for age at menarche (rs7138803, 848 kb apart, *r*^2^=0.02). *PRKAG1* encodes the gamma-1 regulatory subunit of AMP-activated protein kinase, which senses and maintains cellular energy homeostasis by promoting fatty-acid oxidation and inhibiting fatty-acid synthesis; *PRKAG1* is overexpressed in ovarian carcinomas^15^ and is somatically mutated in colorectal cancers^16^.

Our second genotyping approach considered X-chromosome GWAS SNPs in up to 76,831 women of European ancestry from the 23andMe study^17^. Imputation was performed against the 1000 Genomes reference, yielding genotype data for ∼266,000 X-chromosome variants (MAF>1%). Two signals, in/near *IGSF1* and *FAAH2*, reached genome-wide significance for association with age at menarche and both associations were confirmed in 39,486 independent women of European ancestry from the deCODE study.

Common variants in and near *IGSF1* were robustly associated with age at menarche (lead SNP: rs762080, MAF=24%; *β*=0.06 years per allele, *P*=9.4 × 10^−13^; Supplementary Fig. 4). *IGSF1* encodes the immunoglobulin superfamily member 1, which is a plasma membrane glycoprotein highly expressed in the pituitary gland and testis. Rare X-linked mutations in *IGSF1* were recently described to cause central hypothyroidism, hypoprolactinemia, delayed puberty and macro-orchidism in males (OMIM no. 300888)^18^,^19^. Heterozygous female carriers reportedly had normal age at menarche; however, 6/18 had central hypothyroidism and 4/18 underwent oophorectomy for ovarian cysts^19^.

The second X-chromosome locus, in Xp11.21 (lead SNP rs5914101 is intronic in *FAAH2*, MAF 24%, *β*=0.05 years per allele, *P*=1.9 × 10^−10^; Supplementary Fig. 4), lies within the critical region for Turner’s syndrome, which is the most common cause of primary ovarian insufficiency^20^. *FAAH2* encodes fatty-acid amide hydrolase 2. This enzyme catalyses the hydrolysis and degradation of bioactive fatty-acid amides, a large class of endogenous signalling lipids including the endocannabinoids, which modulate several physiological processes, including feeding, inflammation, pain, sleep and various reproductive processes, including hypothalamic gonadotropin-releasing hormone secretion^21^,^22^.

We sought to further functionally characterize the seven genes implicated by these analyses using expression data on 53 tissue types from the Genotype-Tissue Expression consortium^23^. All seven genes showed high relative tissue expression in the ovary and/or brain (specifically the hypothalamus; Supplementary Fig. 5); however, none of the lead SNPs showed a significant association with mRNA transcript abundance. None of the identified variants were associated with body mass index in 74,071 adults from the deCODE study (all *P*>0.05), indicating that their effects on puberty timing are unlikely to be mediated by body mass index.

## Discussion

In summary, by large-scale analysis of genetic variation not captured by previous GWAS for age at menarche, we identified several low-frequency exonic variants of relatively large effect and two common X-chromosome signals. The implicated genes provide new insights into the mechanisms that link energy homeostasis to puberty timing, indicate possible roles of RNA-mediated gene silencing and fatty-acid amide signalling, and link genes behind rare autosomal, X-linked and syndromic disorders of puberty to normal variation in reproductive timing. Our findings using dense exome arrays in large unselected populations are informative for the clinical interpretation of heterozygous *TACR3* variants in patients with rare disorders. In the deCODE study these novel variants collectively explained only 0.5% of the variance in age at menarche, suggesting that these often overlooked sources of genetic variation do not contribute disproportionately to the missing heritability of this complex trait. While variants with MAF below 1% are likely not well represented here, our findings indicate that, similar to other complex traits^24^, the genetic architecture of puberty timing is likely dominated by the additive effects of hundreds or even thousands of variants, each with relatively small effect.

## Methods

### Exome array discovery analysis

Exome array genotype data were generated across 19 studies in up to 76,657 women of genetically determined European ancestry with questionnaire-reported age at menarche between ages 9 and 17 years (Supplementary Table 1). Exome array genotype calling for three studies (Framingham Heart Study (FHS), the Atherosclerosis Risk in Communities (ARIC) and Rotterdam Study (RS); totalling ∼9,000 women) was performed jointly as part of the CHARGE joint calling protocol^25^, which included over 62,000 individuals. Four additional studies (Cambridge Cancer, KORA, Korcula, Generation Scotland, total *N*∼9,700) used the cluster file made available by CHARGE to call genotypes. Other studies followed standard calling and QC protocols for the Exome array (Supplementary Table 2). Each contributing study ran a linear regression model on age at menarche, adjusted for birth year and principal components derived from genotypes, using the skatMeta/seqMeta package in R. Studies with family data included a random effect to account for relationships. Alleles were aligned to a common reference file before association testing (SNPInfo_HumanExome-12v1_rev5.tsv.txt available at http://www.chargeconsortium.com/main/exomechip/) and variants with MAF>5% in the meta-analysis were excluded. We performed gene-based testing (within seqMeta) for low-frequency variants using fixed effect burden tests, which assume that all rare variants have the same effect direction and size (scaled by a weight determined by allele frequency), and SKAT tests, which assume that rare variant effects are random and can contain a mixture of null, protective and risk rare alleles. These tests were run using three variant filters, all of which included only variants with MAF<1%: (1) all non-synonymous; (2) non-synonymous annotated as ‘damaging’ (conserved and predicted damaging, see http://www.chargeconsortium.com/main/exomechip/); and (3) only loss of function. The multiple testing adjustment included two tests × three filters × number of genes, requiring study-wise significance threshold *P*<1.14 × 10^−6^. For individual variants, a fixed-effects inverse variance-weighted meta-analysis was performed across all studies using METAL (http://www.sph.umich.edu/csg/abecasis/Metal/), with associations considered significant at a conservative genome-wide significance threshold of *P*<5 × 10^−8^.

### Exome array follow-up studies

We performed follow-up testing of selected exome array variants in the 23andMe study (as described below) and also in 39,486 independent women of European ancestry from the deCODE study, Iceland, who had genotypes on over 34 million variants by imputation of whole-genome sequencing-identified SNPs and indels on Illumina SNP chip data (Supplementary Table 1)^26^. Variants from both studies were required to either pass genotyping QC (23andMe only, described below) or have imputation quality score >0.4. X-chromosome follow-up was performed in the deCODE study alone. All participants in all published studies provided informed consent, and the research protocol of each study was approved by their local research ethics committee^1^.

### 1000G X-chromosome discovery meta-analysis

X-chromosome SNP data were generated in up to 76,831 women of European ancestry from the 23andMe study^17^,^27^, with questionnaire-reported age at menarche between the ages of 8 and 16 years, and who were genotyped on one or more of three GWAS arrays that also included customized content on human pathogenic variants (Supplementary Table 1)^28^. 23andMe participants provided informed consent to take part in this research under a protocol approved by the AAHRPP-accredited institutional review board, Ethical and Independent Review Services. Before imputation, we excluded SNPs with Hardy–Weinberg equilibrium *P*<10^−20^, call rate <95% or with large allele frequency discrepancies compared with European 1000 Genomes reference data. Frequency discrepancies were identified by computing a 2 × 2 table of allele counts for European 1000 Genomes samples and 2,000 randomly sampled 23andMe customers with European ancestry, and identifying SNPs with a *χ*^2^ *P*<10^−15^. Genotype data were imputed against the March 2012 ‘v3’ release of 1000 Genomes reference haplotypes. Age at menarche was assessed by questionnaire and recorded in 2-year-age bins, which were rescaled to 1-year effect estimates post analysis. The validity of this approach was confirmed by the lack of significant heterogeneity between rescaled 23andMe menarche estimates for the 123 previously identified signals and their reported effects^1^. Association results were obtained from linear regression models assuming additive allelic effects. These models included covariates for age and the top five GWAS SNP principal components to account for residual population structure. Results were further adjusted for a lambda genomic control value of 1.152 to correct for any residual test statistic inflation due to population stratification. Linkage disequilibrium score regression analysis (LDSC)^29^ confirmed that principle component correction appropriately controlled for potential test statistic inflation due to population stratification (pre-genomic control-corrected calculated intercept ∼1). The reported association test *P* values were computed from likelihood ratio tests.

### X-chromosome follow-up

Identified X-chromosome variants were replicated in 39,486 women from the deCODE study, as described above.

## Additional information

**How to cite this article:** Lunetta, K. L. *et al*. Rare coding variants and X-linked loci associated with age at menarche. *Nat. Commun*. 6:7756 doi: 10.1038/ncomms8756 (2015).

## Supplementary Material

Supplementary Information Supplementary Figures 1-5 and Supplementary Tables 1-3

## Figures and Tables

**Table 1:** Association statistics for the novel low-frequency and X-chromosome variants.

**Gene**	**SNP**	**Location**	**Alleles** *	**Discovery**				**Follow-up**			**Combined**		
				**Effect (s.e.)**		***P***	***N***	**Effect (s.e.) | VE** †	***P***	***N***	**Effect (s.e.)**	***P***	***N***
*Exome array*													
*ALMS1*	rs45501594	2p13.1	G/C/1.1%		0.26 (0.04)	4.6E−10	57,867	0.23 (0.03) | 0.12%	2.2E–11	116,317	0.24 (0.03)	6.8E–20	174,184
*LAMB2*	rs35713889	3p21.31	T/C/4.4%		0.11 (0.02)	5.0E−07	58,695	0.08 (0.02) | 0.04%	2.2E–06	116,317	0.09 (0.01)	1.0E–11	175,012
*TNRC6A*	rs113388806	16p12.1	T/A/4.7%		0.09 (0.02)	1.7E−05	76,657	0.08 (0.02) | 0.04%	1.4E–07	116,317	0.08 (0.01)	1.1E–11	192,974
*TACR3*	rs144292455	4q24	T/C/0.08%		0.71 (0.15)	1.3E−06	68,487	1.25 (0.25) | 0.20%	8.0E–07	116,317	0.84 (0.13)	2.8E–11	184,804
*PRKAG1*	rs1126930	12q13.12	C/G/3.4%		−0.11 (0.02)	4.4E−07	76,657	−0.08 (0.02) | 0.02%	3.6E–05	116,317	−0.09 (0.01)	9.6E–11	192,974
													
*1000G X-chromosome*													
*IGSF1*	rs762080	Xq26.2	A/C/24%		−0.07 (0.01)	4.1E−12	76,831	−0.04 (0.01) | 0.04%	6.7E–03	39,486	−0.06 (0.008)	9.4E–13	116,317
*FAAH2*	rs5914101	Xp11.21	A/G/24%		−0.07 (0.01)	1.1E−09	76,831	−0.03 (0.01) | 0.03%	2.0E–02	39,486	−0.05 (0.009)	4.9E–10	116,317

deCODE, Diabetes Epidemiology: Collaborative analysis of Diagnostic criteria in Europe; SNP, single-nucleotide polymorphism; VE, variance explained.

^*^Refers to effect allele/other allele/effect allele frequency.

^†^Beta (standard error) from the combined replication samples | VE in the deCODE study. Units are on a 1-year scale.
