# Corrigendum: Rare coding variants and X-linked loci associated with age at menarche

**DOI:** 10.1038/ncomms10257

**Published:** 2015-12-17

**Authors:** Kathryn L. Lunetta, Felix R. Day, Patrick Sulem, Katherine S. Ruth, Joyce Y. Tung, David A. Hinds, Tõnu Esko, Cathy E. Elks, Elisabeth Altmaier, Chunyan He, Jennifer E. Huffman, Evelin Mihailov, Eleonora Porcu, Antonietta Robino, Lynda M. Rose, Ursula M. Schick, Lisette Stolk, Alexander Teumer, Deborah J. Thompson, Michela Traglia, Carol A. Wang, Laura M. Yerges-Armstrong, Antonis C. Antoniou, Caterina Barbieri, Andrea D. Coviello, Francesco Cucca, Ellen W. Demerath, Alison M. Dunning, Ilaria Gandin, Megan L. Grove, Daniel F. Gudbjartsson, Lynne J. Hocking, Albert Hofman, Jinyan Huang, Rebecca D. Jackson, David Karasik, Jennifer Kriebel, Ethan M. Lange, Leslie A. Lange, Claudia Langenberg, Xin Li, Jian'an Luan, Reedik Mägi, Alanna C. Morrison, Sandosh Padmanabhan, Ailith Pirie, Ozren Polasek, David Porteous, Alex P. Reiner, Fernando Rivadeneira, Igor Rudan, Cinzia F. Sala, David Schlessinger, Robert A. Scott, Doris Stöckl, Jenny A. Visser, Uwe Völker, Diego Vozzi, James G. Wilson, Marek Zygmunt, Eric Boerwinkle, Julie E. Buring, Laura Crisponi, Douglas F. Easton, Caroline Hayward, Frank B. Hu, Simin Liu, Andres Metspalu, Craig E. Pennell, Paul M. Ridker, Konstantin Strauch, Elizabeth A. Streeten, Daniela Toniolo, André G. Uitterlinden, Sheila Ulivi, Henry Völzke, Nicholas J. Wareham, Melissa Wellons, Nora Franceschini, Daniel I. Chasman, Unnur Thorsteinsdottir, Anna Murray, Kari Stefansson, Joanne M. Murabito, Ken K. Ong, John R. B. Perry

Nature Communications 6, Article number: 7756 10.1038/ncomms8756 (2015); Published 08042015; Updated 12172016

In the Results section and in the legend of Table 1 of this Article, the company deCODE genetics, Inc. is incorrectly referred to as ‘Diabetes Epidemiology: collaborative analysis of Diagnostic criteria in Europe'. In neither instance is deCODE an abbreviation.

