# Supplementary material for: Rare coding variants and X-linked loci associated with age at menarche
Source: Nat Commun. 2015 Aug 4;6:7756. doi: 10.1038/ncomms8756 (PMC4538850; doi:10.1038/ncomms8756)
Supplement: Supplementary Information — Supplementary Figures 1-5 and Supplementary Tables 1-3 [file ncomms8756-s1.pdf]

Supplementary Figure 1 | Quantile-Quantile plot of low-frequency exome array variants showing the expected vs observed distribution of test statistic with age at menarche

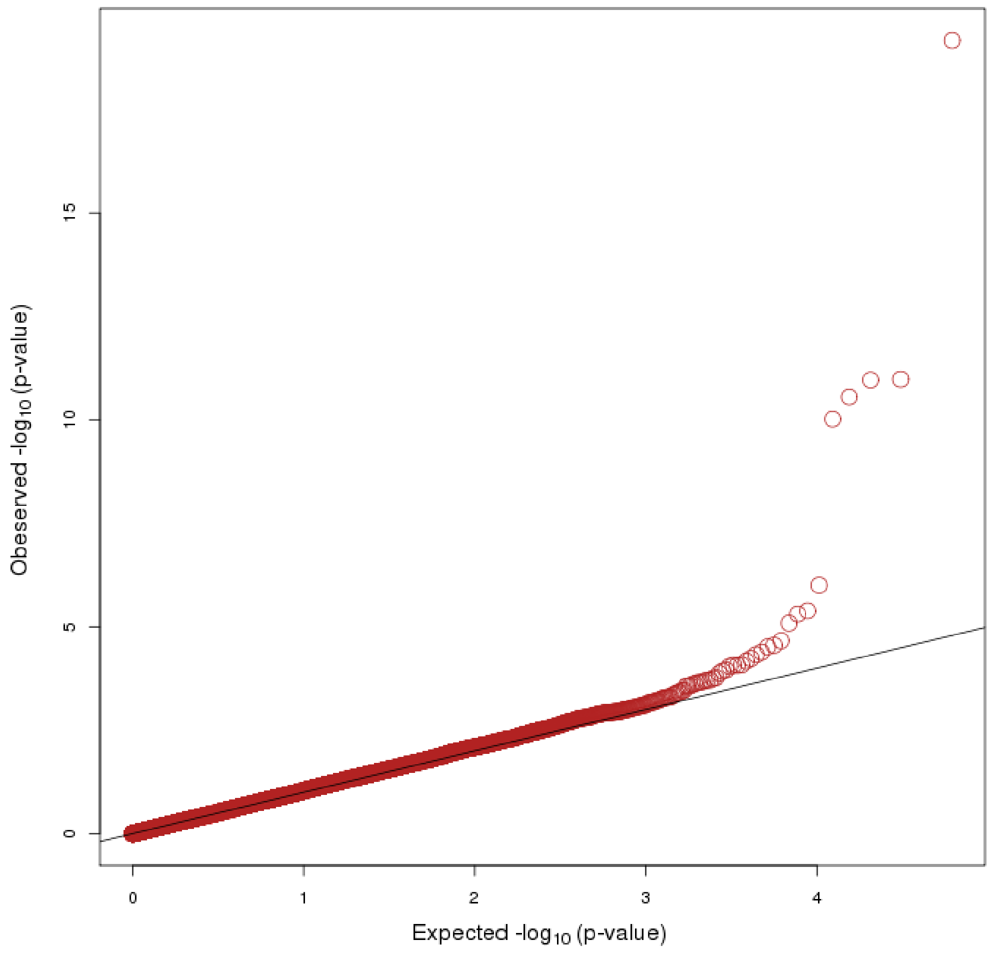

Supplementary Figures 2A-E | Forest plots for significantly associated low-frequency exome array variants for genes a) *ALMS1*, b) *LAMB2*, c) *TNR6CA*, d) *TACR3*, e) *PRKAG1*

A

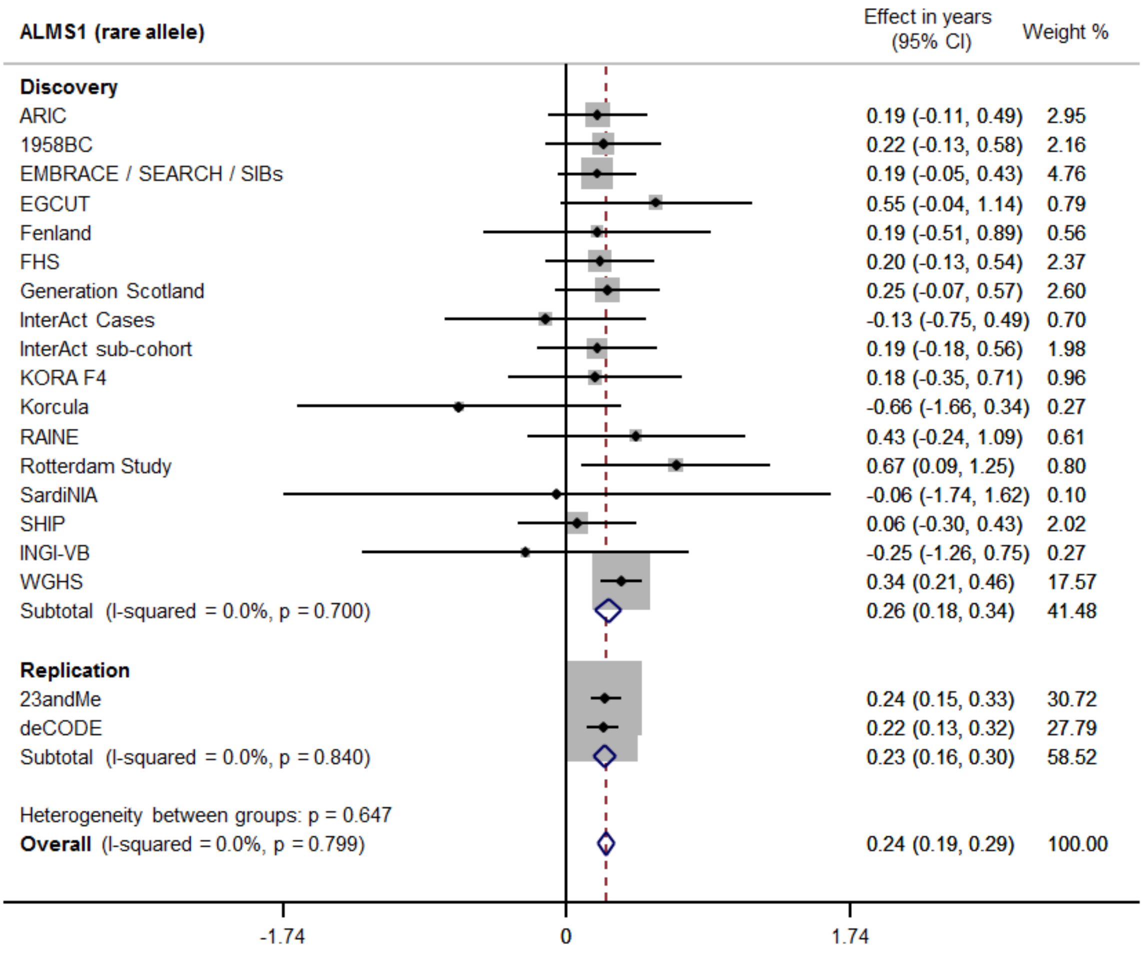

B

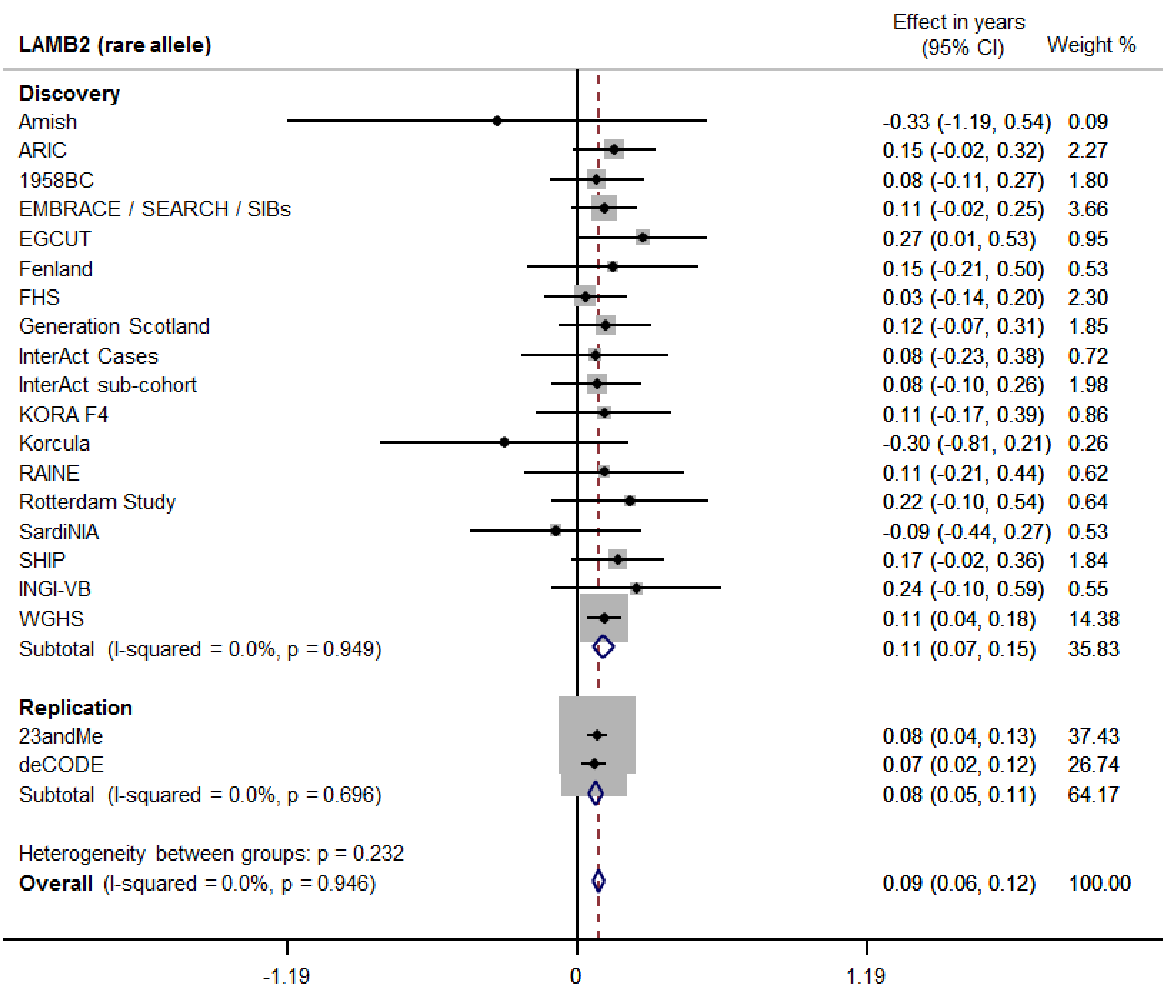
LAMB2 (rare allele)
Effect in years (95% CI)
Weight %
Discovery
Amish -0.33 (-1.19, 0.54) 0.09
ARIC 0.15 (-0.02, 0.32) 2.27
1958BC 0.08 (-0.11, 0.27) 1.80
EMBRACE / SEARCH / SIBs 0.11 (-0.02, 0.25) 3.66
EGCUT 0.27 (0.01, 0.53) 0.95
Fenland 0.15 (-0.21, 0.50) 0.53
FHS 0.03 (-0.14, 0.20) 2.30
Generation Scotland 0.12 (-0.07, 0.31) 1.85
InterAct Cases 0.08 (-0.23, 0.38) 0.72
InterAct sub-cohort 0.08 (-0.10, 0.26) 1.98
KORA F4 0.11 (-0.17, 0.39) 0.86
Korcula -0.30 (-0.81, 0.21) 0.26
RAINE 0.11 (-0.21, 0.44) 0.62
Rotterdam Study 0.22 (-0.10, 0.54) 0.64
SardiNIA -0.09 (-0.44, 0.27) 0.53
SHIP 0.17 (-0.02, 0.36) 1.84
INGI-VB 0.24 (-0.10, 0.59) 0.55
WGHS 0.11 (0.04, 0.18) 14.38
Subtotal (I-squared = 0.0%, p = 0.949) 0.11 (0.07, 0.15) 35.83
Replication
23andMe 0.08 (0.04, 0.13) 37.43
deCODE 0.07 (0.02, 0.12) 26.74
Subtotal (I-squared = 0.0%, p = 0.696) 0.08 (0.05, 0.11) 64.17
Heterogeneity between groups: p = 0.232
Overall (I-squared = 0.0%, p = 0.946) 0.09 (0.06, 0.12) 100.00
-1.19
0
1.19

C

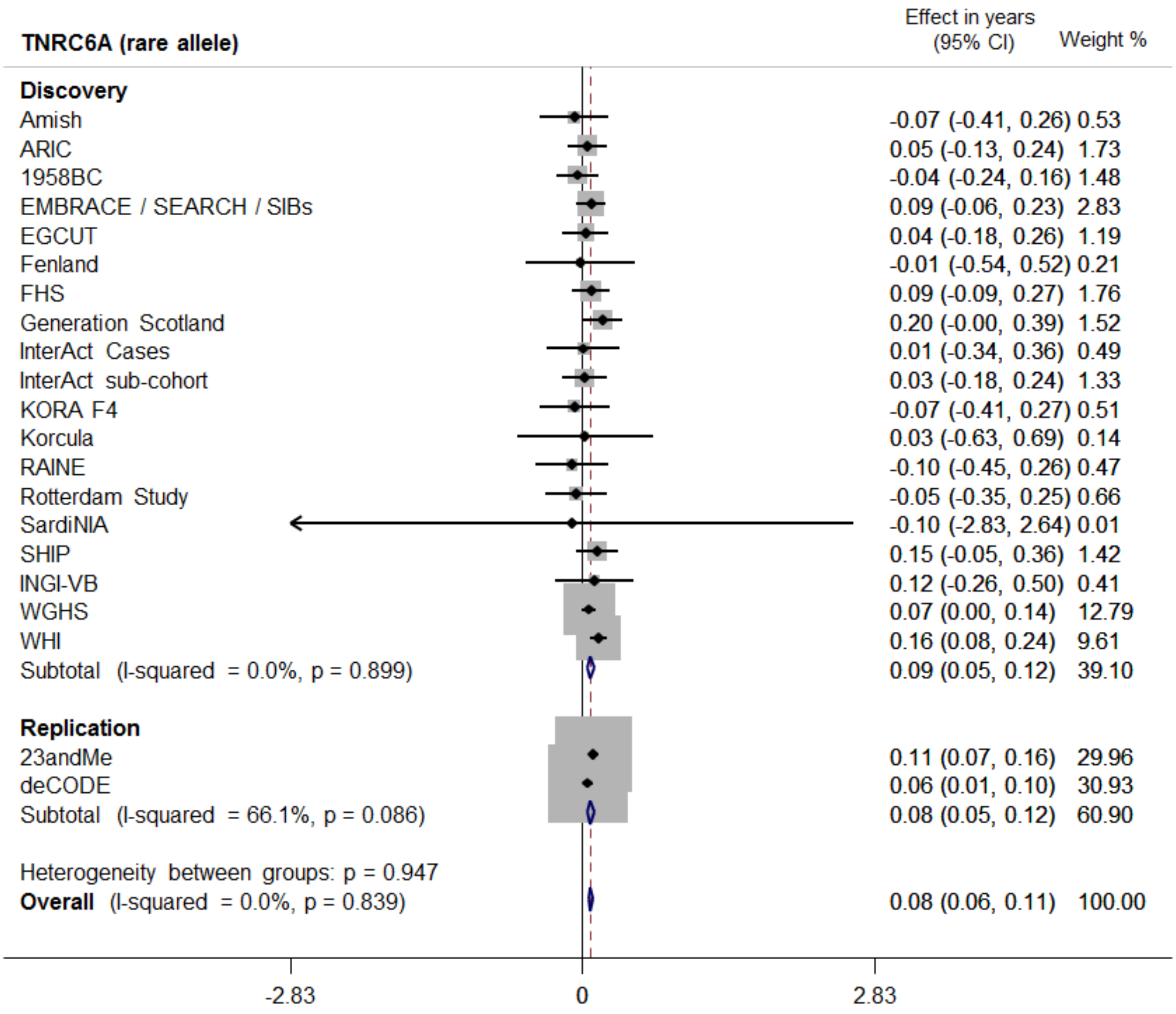
TNRC6A (rare allele)
Effect in years (95% CI)
Weight %
Discovery
Amish -0.07 (-0.41, 0.26) 0.53
ARIC 0.05 (-0.13, 0.24) 1.73
1958BC -0.04 (-0.24, 0.16) 1.48
EMBRACE / SEARCH / SIBs 0.09 (-0.06, 0.23) 2.83
EGCUT 0.04 (-0.18, 0.26) 1.19
Fenland -0.01 (-0.54, 0.52) 0.21
FHS 0.09 (-0.09, 0.27) 1.76
Generation Scotland 0.20 (-0.00, 0.39) 1.52
InterAct Cases 0.01 (-0.34, 0.36) 0.49
InterAct sub-cohort 0.03 (-0.18, 0.24) 1.33
KORA F4 -0.07 (-0.41, 0.27) 0.51
Korcula 0.03 (-0.63, 0.69) 0.14
RAINE -0.10 (-0.45, 0.26) 0.47
Rotterdam Study -0.05 (-0.35, 0.25) 0.66
SardiNIA -0.10 (-2.83, 2.64) 0.01
SHIP 0.15 (-0.05, 0.36) 1.42
INGI-VB 0.12 (-0.26, 0.50) 0.41
WGHS 0.07 (0.00, 0.14) 12.79
WHI 0.16 (0.08, 0.24) 9.61
Subtotal (I-squared = 0.0%, p = 0.899) 0.09 (0.05, 0.12) 39.10
Replication
23andMe 0.11 (0.07, 0.16) 29.96
deCODE 0.06 (0.01, 0.10) 30.93
Subtotal (I-squared = 66.1%, p = 0.086) 0.08 (0.05, 0.12) 60.90
Heterogeneity between groups: p = 0.947
Overall (I-squared = 0.0%, p = 0.839) 0.08 (0.06, 0.11) 100.00
-2.83
0
2.83

D.

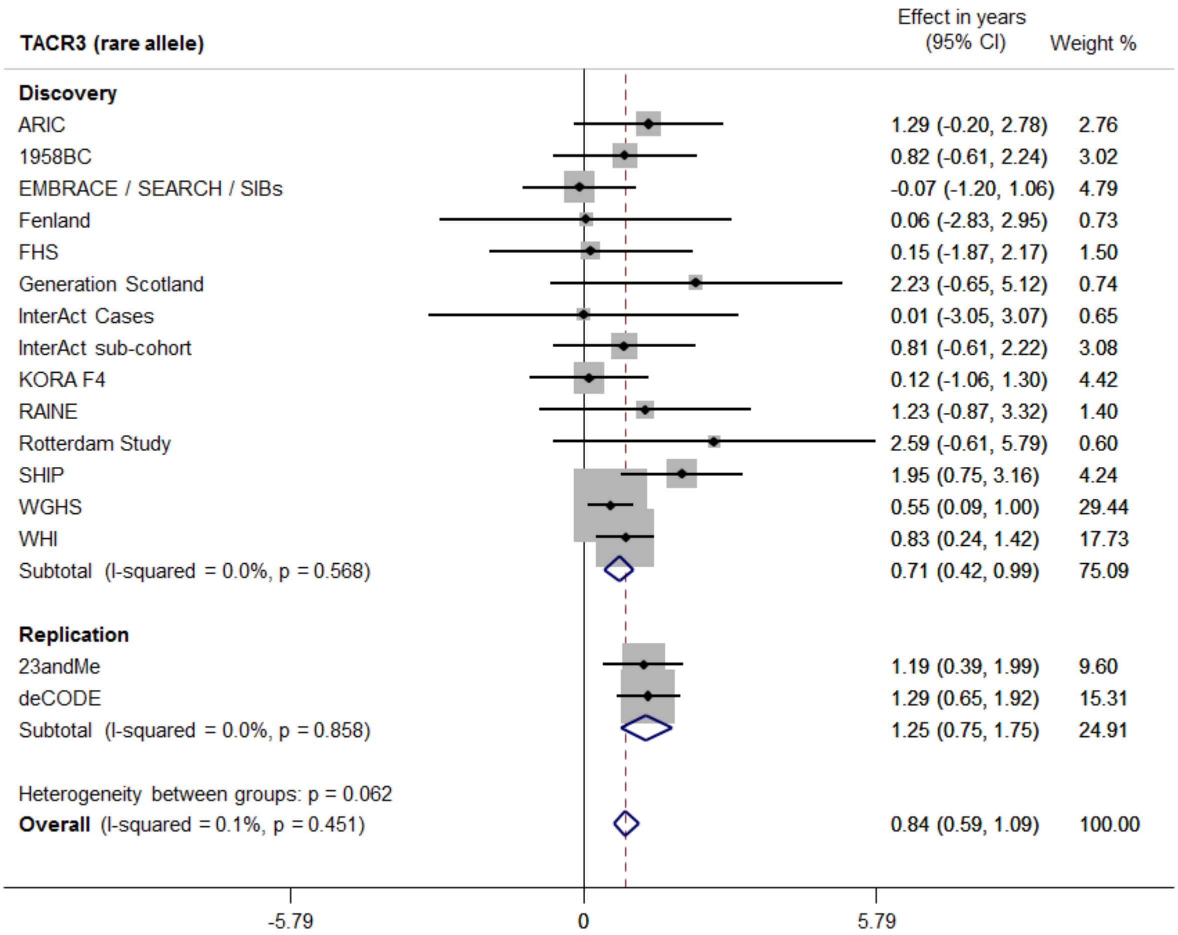
TACR3 (rare allele)
Effect in years
(95% CI)
Weight %
Discovery
ARIC
1.29 (-0.20, 2.78)
2.76
1958BC
0.82 (-0.61, 2.24)
3.02
EMBRACE / SEARCH / SIBs
-0.07 (-1.20, 1.06)
4.79
Fenland
0.06 (-2.83, 2.95)
0.73
FHS
0.15 (-1.87, 2.17)
1.50
Generation Scotland
2.23 (-0.65, 5.12)
0.74
InterAct Cases
0.01 (-3.05, 3.07)
0.65
InterAct sub-cohort
0.81 (-0.61, 2.22)
3.08
KORA F4
0.12 (-1.06, 1.30)
4.42
RAINE
1.23 (-0.87, 3.32)
1.40
Rotterdam Study
2.59 (-0.61, 5.79)
0.60
SHIP
1.95 (0.75, 3.16)
4.24
WGHS
0.55 (0.09, 1.00)
29.44
WHI
0.83 (0.24, 1.42)
17.73
Subtotal (I-squared = 0.0%, p = 0.568)
0.71 (0.42, 0.99)
75.09
Replication
23andMe
1.19 (0.39, 1.99)
9.60
deCODE
1.29 (0.65, 1.92)
15.31
Subtotal (I-squared = 0.0%, p = 0.858)
1.25 (0.75, 1.75)
24.91
Heterogeneity between groups: p = 0.062
Overall (I-squared = 0.1%, p = 0.451)
0.84 (0.59, 1.09)
100.00
-5.79
0
5.79

E.

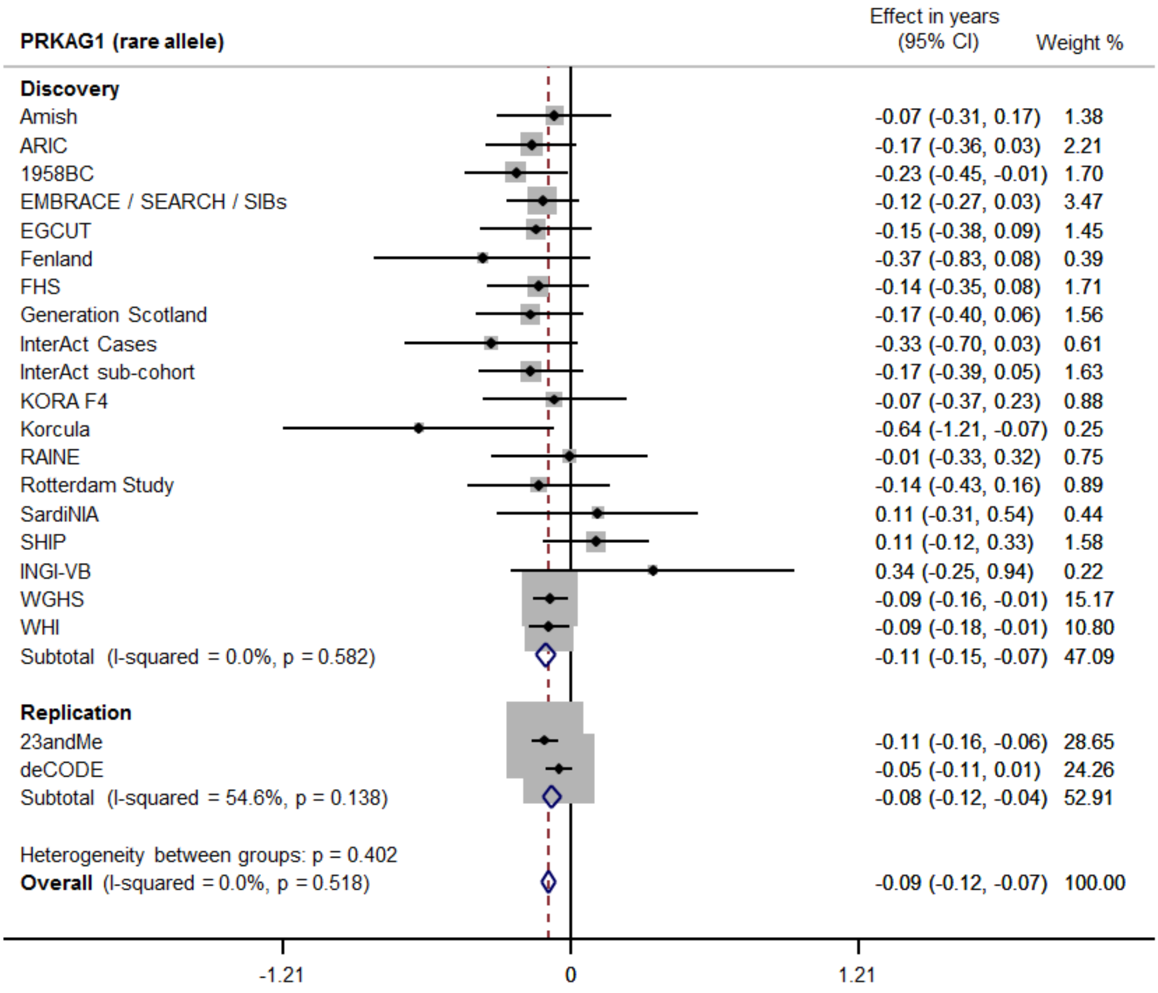
PRKAG1 (rare allele)
Effect in years (95% CI)
Weight %
Discovery
Amish -0.07 (-0.31, 0.17) 1.38
ARIC -0.17 (-0.36, 0.03) 2.21
1958BC -0.23 (-0.45, -0.01) 1.70
EMBRACE / SEARCH / SIBs -0.12 (-0.27, 0.03) 3.47
EGCUT -0.15 (-0.38, 0.09) 1.45
Fenland -0.37 (-0.83, 0.08) 0.39
FHS -0.14 (-0.35, 0.08) 1.71
Generation Scotland -0.17 (-0.40, 0.06) 1.56
InterAct Cases -0.33 (-0.70, 0.03) 0.61
InterAct sub-cohort -0.17 (-0.39, 0.05) 1.63
KORA F4 -0.07 (-0.37, 0.23) 0.88
Korcula -0.64 (-1.21, -0.07) 0.25
RAINE -0.01 (-0.33, 0.32) 0.75
Rotterdam Study -0.14 (-0.43, 0.16) 0.89
SardiNIA 0.11 (-0.31, 0.54) 0.44
SHIP 0.11 (-0.12, 0.33) 1.58
INGI-VB 0.34 (-0.25, 0.94) 0.22
WGHS -0.09 (-0.16, -0.01) 15.17
WHI -0.09 (-0.18, -0.01) 10.80
Subtotal (I-squared = 0.0%, p = 0.582) -0.11 (-0.15, -0.07) 47.09
Replication
23andMe -0.11 (-0.16, -0.06) 28.65
deCODE -0.05 (-0.11, 0.01) 24.26
Subtotal (I-squared = 54.6%, p = 0.138) -0.08 (-0.12, -0.04) 52.91
Heterogeneity between groups: p = 0.402
Overall (I-squared = 0.0%, p = 0.518) -0.09 (-0.12, -0.07) 100.00
-1.21
0
1.21

Supplementary Figure 3A-E| Regional association plots of the regions highlighted in the exome analysis showing the region around the signal for: A) *ALMS1* on chromosome 2, B) *LAMB2* on chromosome 3, C) *TACR3* on chromosome 4, D) *PRKAG1* on chromosome 12, E) *TNRC6A* on chromosome 16.

A

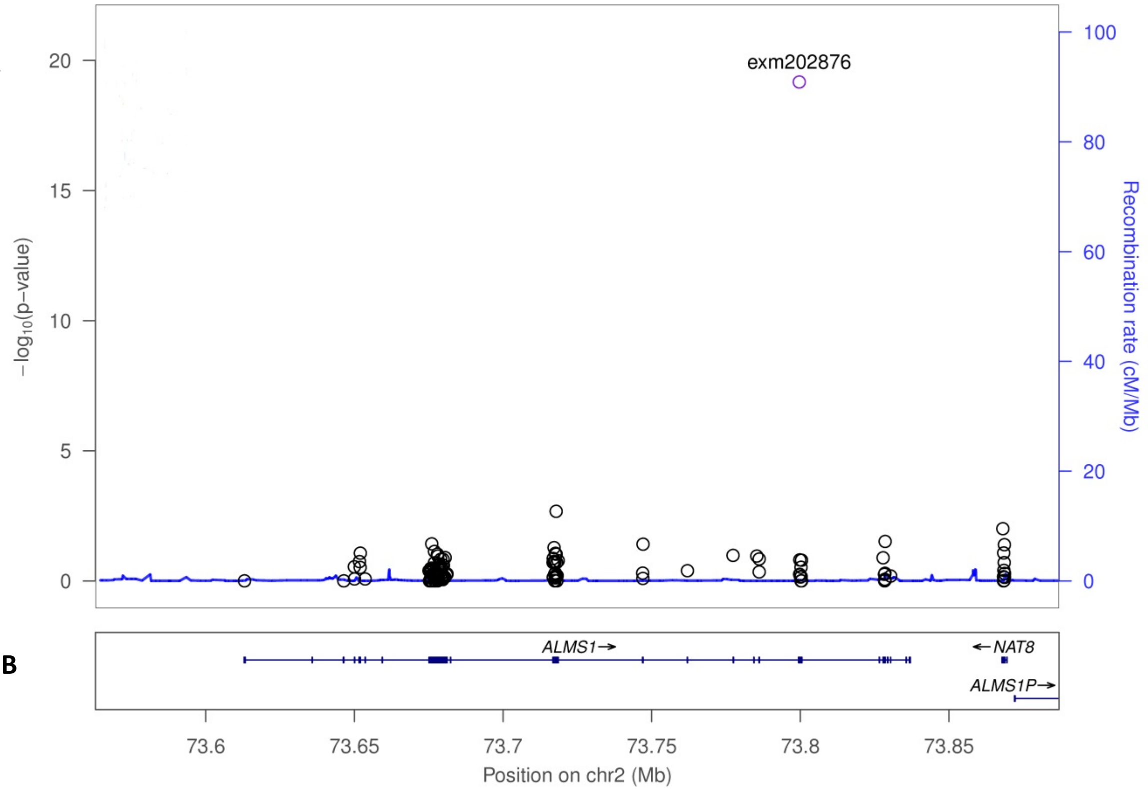

B

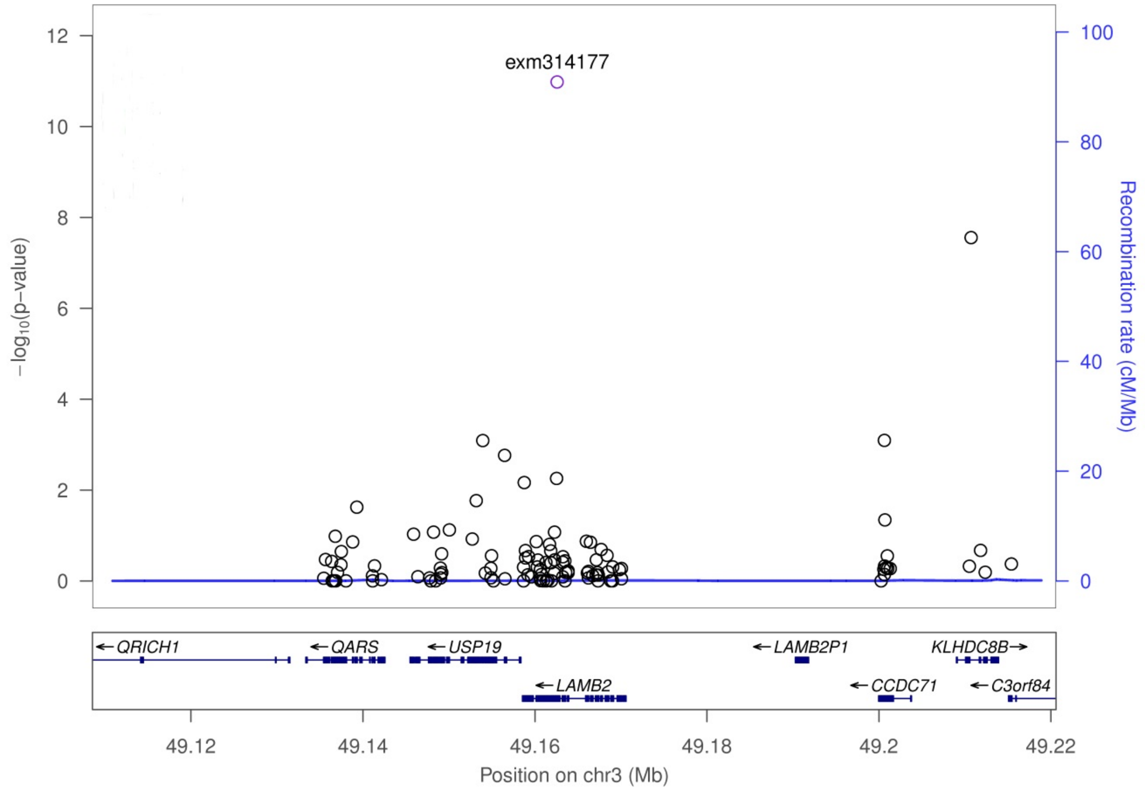

C

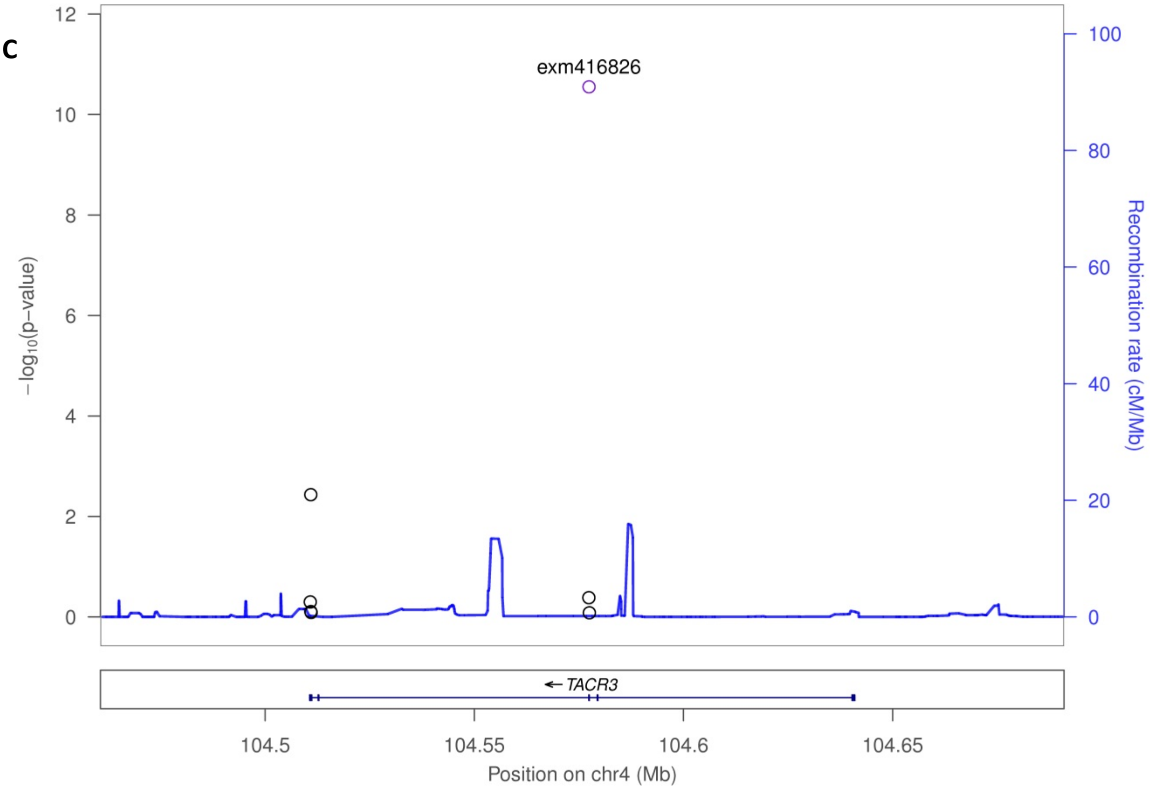
exm416826
−log10(p−value)
Recombination rate (cM/Mb)
TACR3
104.5
104.55
104.6
104.65
Position on chr4 (Mb)

D

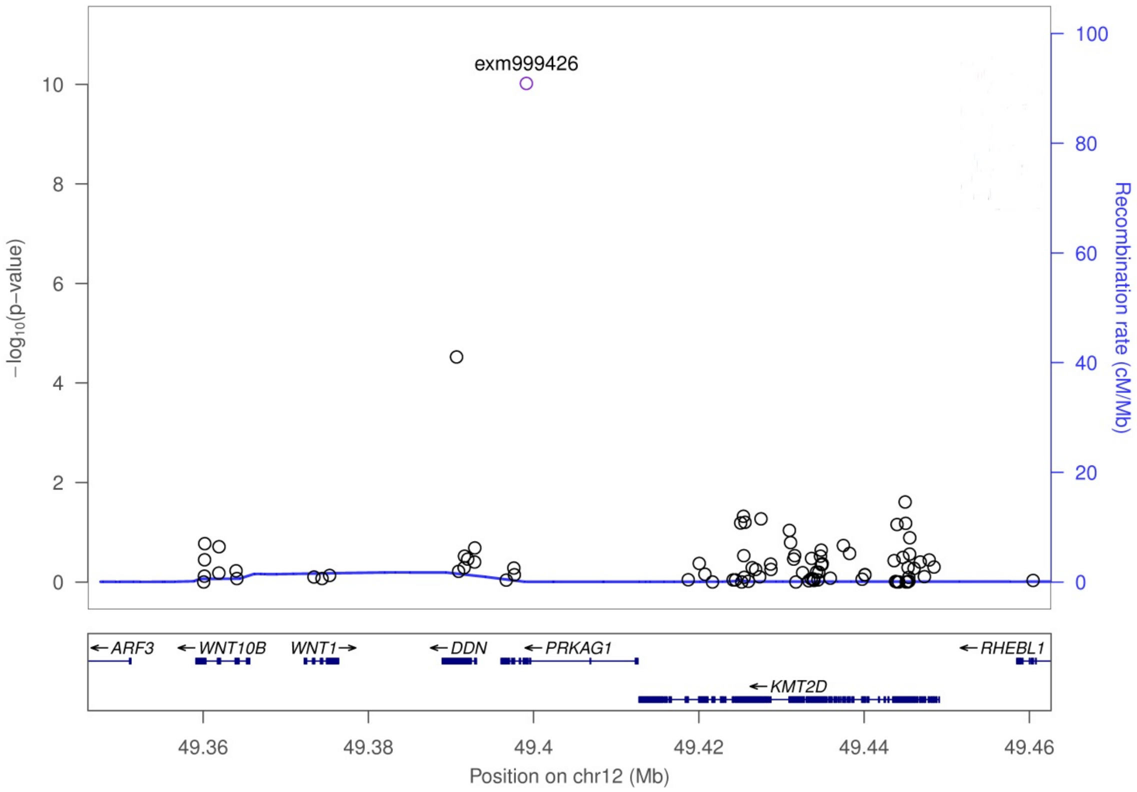
exm999426
−log10(p−value)
Recombination rate (cM/Mb)
ARF3
WNT10B
WNT1
DDN
PRKAG1
KMT2D
RHEBL1
49.36
49.38
49.4
49.42
49.44
49.46
Position on chr12 (Mb)

E

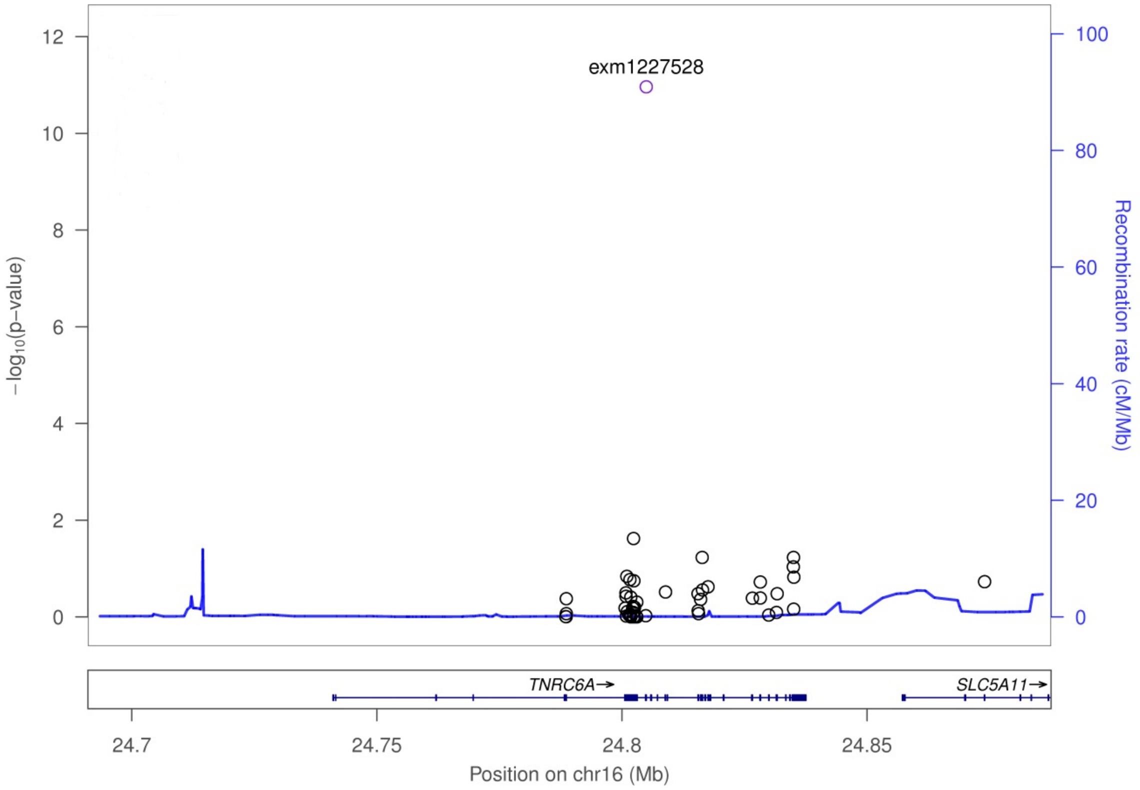

exm1227528
12
10
8
6
4
2
0
−log10(p−value)
100
80
60
40
20
0
Recombination rate (cM/Mb)
TNRC6A→
SLC5A11→
24.7
24.75
24.8
24.85
Position on chr16 (Mb)

Supplementary Figure 4 | Regional association plots of the two common signals found on the X-chromosome: A) the signal at rs762080 near to *IGSF1*, and B) the signal at rs5914101 in *FAAH2*. In each case the surrounding SNPs are coloured to show levels of linkage disequilibrium with the SNP with the lowest p-value.

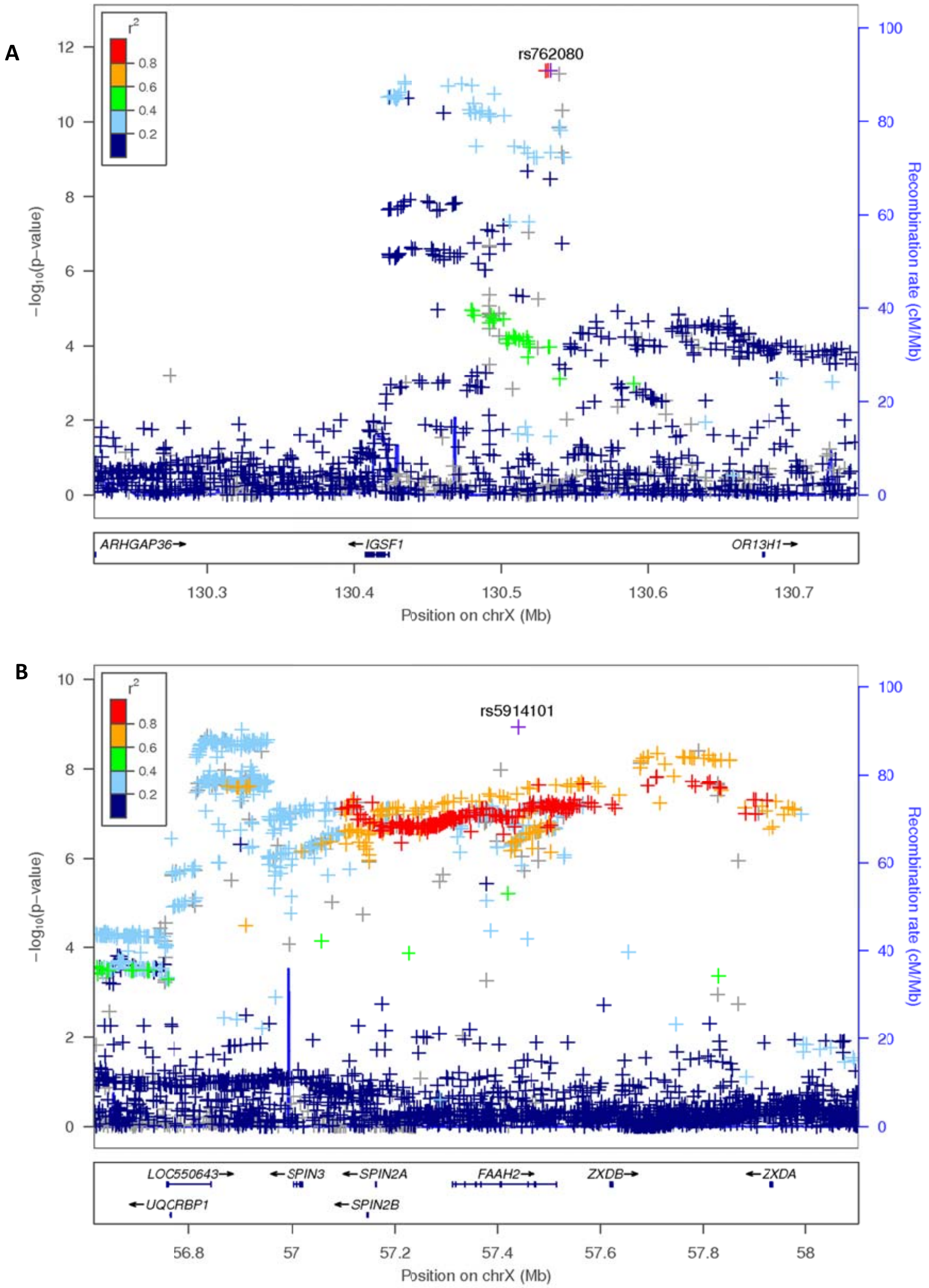

Supplementary Figure 5 | Relative gene expression profiles of highlighted genes using data from the GTex Consortium

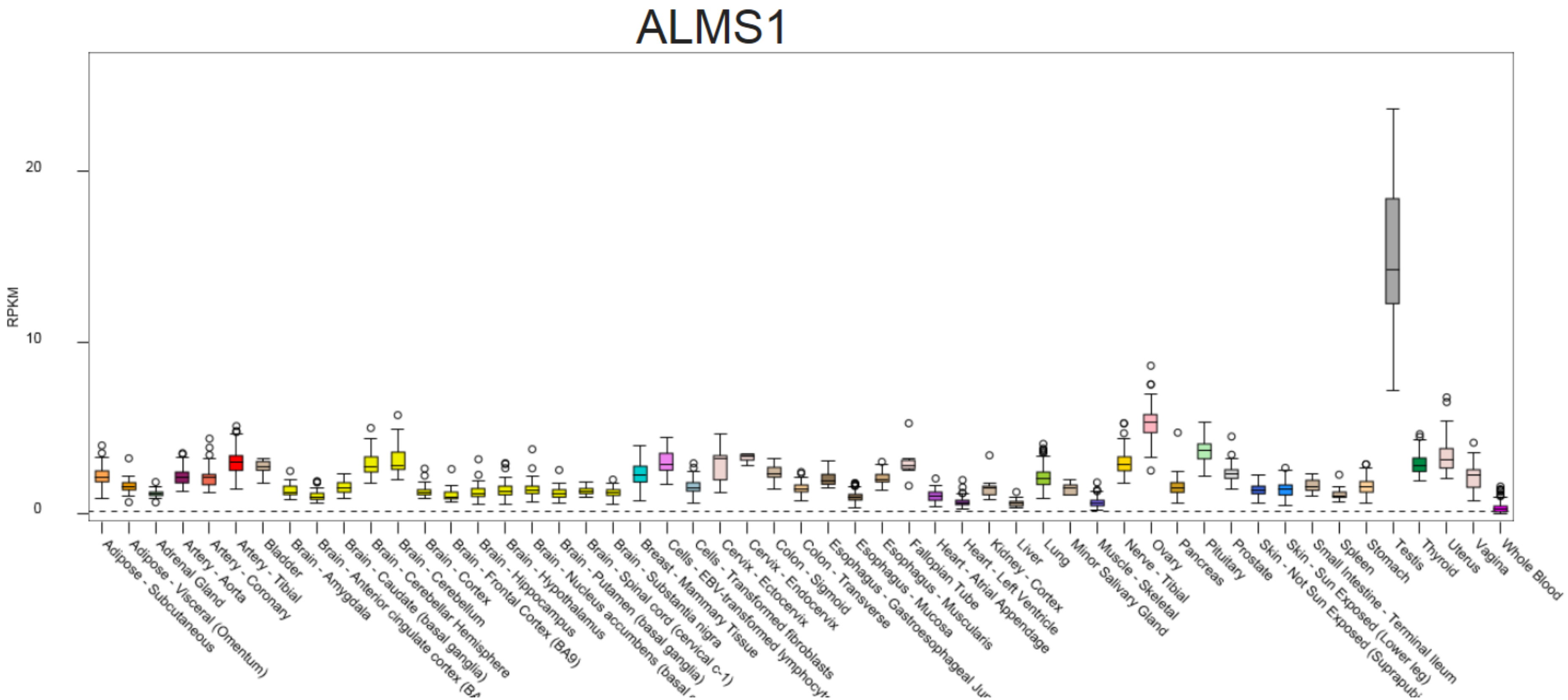

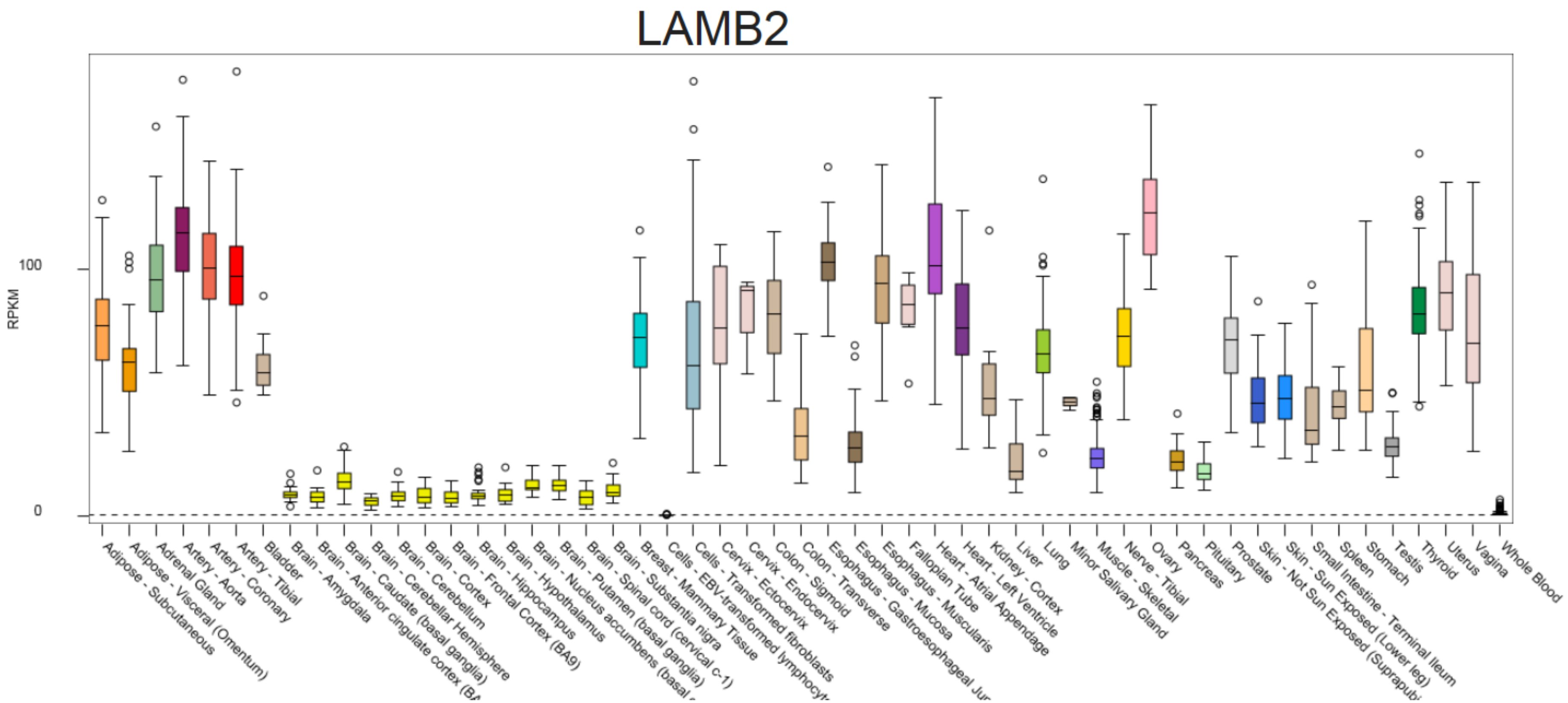
LAMB2
RPKM
100
0
Adipose - Subcutaneous
Adipose - Visceral (Omentum)
Adrenal Gland
Artery - Aorta
Artery - Coronary
Artery - Tibial
Bladder
Brain - Amygdala
Brain - Caudate (basal ganglia)
Brain - Cerebellar Hemisphere
Brain - Cerebellum
Brain - Cortex
Brain - Frontal Cortex (BA9)
Brain - Hippocampus
Brain - Hypothalamus
Brain - Putamen (basal ganglia)
Brain - Spinal cord (cervical c-1)
Brain - Substantia nigra
Breast - Mammary Tissue
Cells - Transformed fibroblasts
Cervix - Ectocervix
Cervix - Endocervix
Colon - Sigmoid
Colon - Transverse
Esophagus - Mucosa
Esophagus - Muscularis
Fallopian Tube
Heart - Atrial Appendage
Heart - Left Ventricle
Kidney - Cortex
Liver
Lung
Minor Salivary Gland
Muscle - Skeletal
Nerve - Tibial
Ovary
Pancreas
Pituitary
Prostate
Skin - Sun Exposed (Lower leg)
Small Intestine - Terminal Ileum
Spleen
Stomach
Testis
Thyroid
Uterus
Vagina
Whole Blood

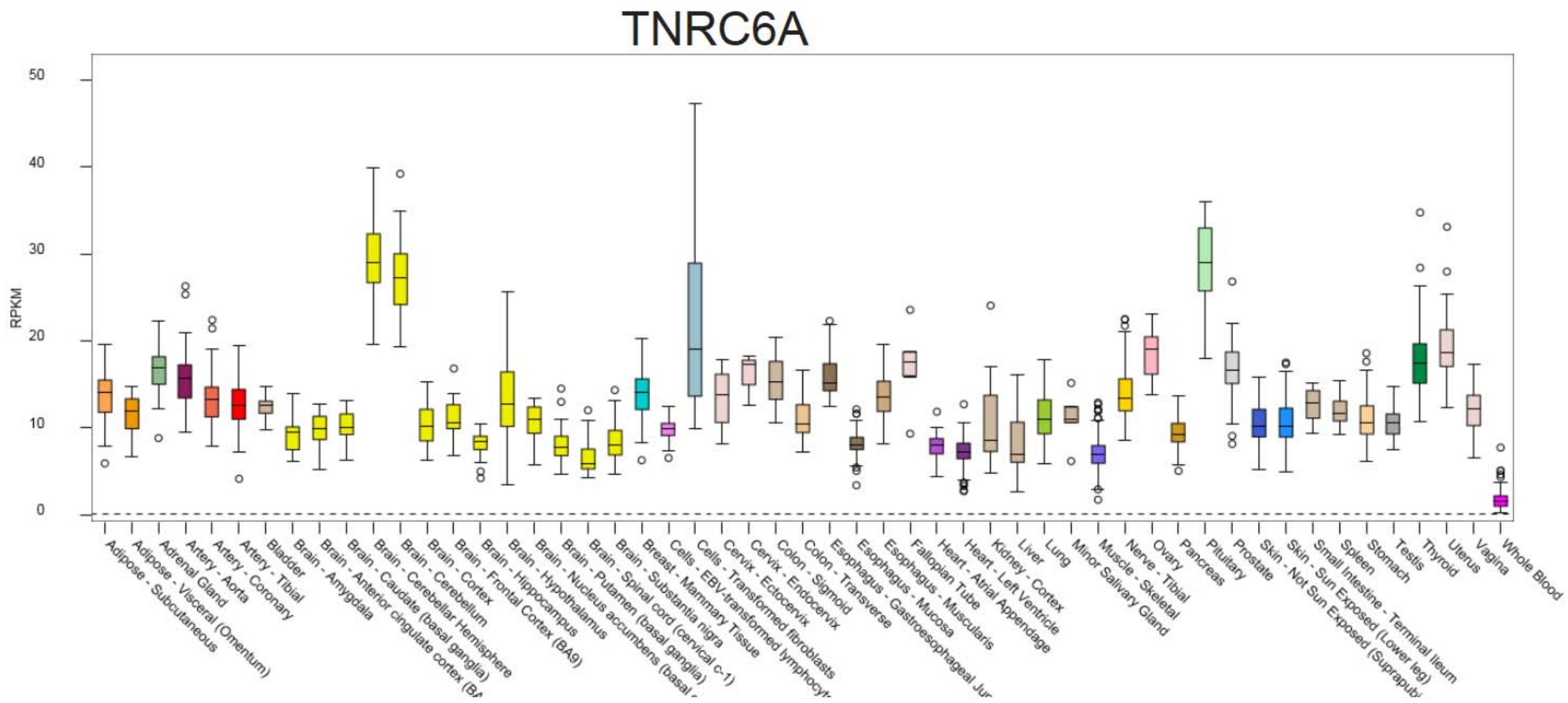
TNRC6A
RPKM
50
40
30
20
10
0
Adipose - Subcutaneous
Adipose - Visceral (Omentum)
Adrenal Gland
Artery - Aorta
Artery - Coronary
Artery - Tibial
Bladder
Brain - Amygdala
Brain - Anterior cingulate cortex (BA
Brain - Caudate (basal ganglia)
Brain - Cerebellar Hemisphere
Brain - Cerebellum
Brain - Cortex
Brain - Frontal Cortex (BA9)
Brain - Hippocampus
Brain - Hypothalamus
Brain - Nucleus accumbens (basal
Brain - Putamen (basal ganglia)
Brain - Spinal cord (cervical c-1)
Brain - Substantia nigra
Breast - Mammary Tissue
Cells - EBV-transformed lymphocyt
Cells - Transformed fibroblasts
Cervix - Ectocervix
Cervix - Endocervix
Colon - Sigmoid
Colon - Transverse
Esophagus - Gastroesophageal Ju
Esophagus - Mucosa
Esophagus - Muscularis
Fallopian Tube
Heart - Atrial Appendage
Heart - Left Ventricle
Kidney - Cortex
Liver
Lung
Minor Salivary Gland
Muscle - Skeletal
Nerve - Tibial
Ovary
Pancreas
Pituitary
Prostate
Skin - Not Sun Exposed (Suprapubi
Skin - Sun Exposed (Lower leg)
Small Intestine - Terminal Ileum
Spleen
Stomach
Testis
Thyroid
Uterus
Vagina
Whole Blood

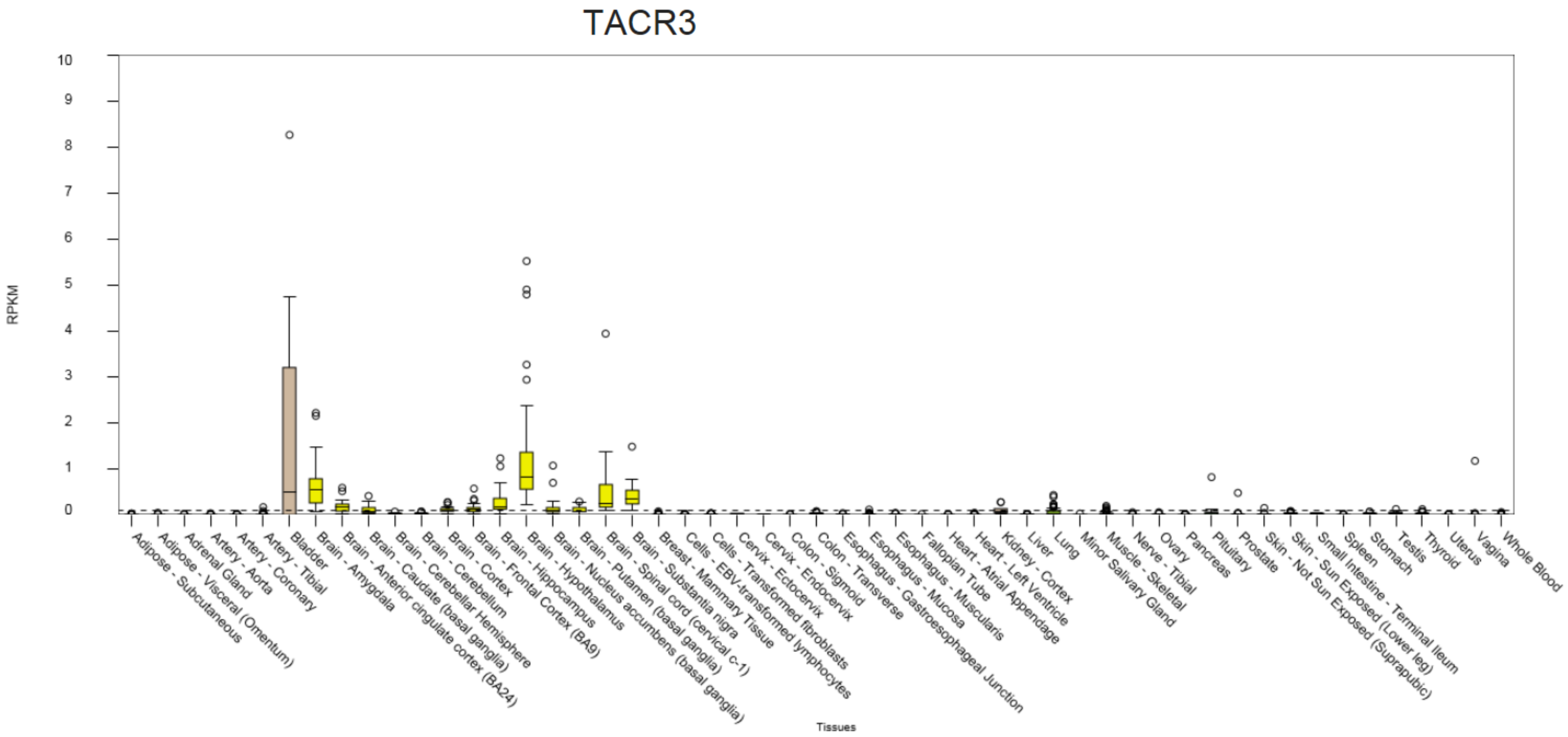
TACR3
RPKM
10
9
8
7
6
5
4
3
2
1
0
Adipose - Subcutaneous
Adipose - Visceral (Omentum)
Adrenal Gland
Artery - Aorta
Artery - Coronary
Artery - Tibial
Bladder
Brain - Amygdala
Brain - Anterior cingulate cortex (BA24)
Brain - Caudate (basal ganglia)
Brain - Cerebellar Hemisphere
Brain - Cerebellum
Brain - Cortex
Brain - Frontal Cortex (BA9)
Brain - Hippocampus
Brain - Hypothalamus
Brain - Nucleus accumbens (basal ganglia)
Brain - Putamen (basal ganglia)
Brain - Spinal cord (cervical c-1)
Brain - Substantia nigra
Breast - Mammary Tissue
Cells - EBV-transformed lymphocytes
Cells - Transformed fibroblasts
Cervix - Ectocervix
Cervix - Endocervix
Colon - Sigmoid
Colon - Transverse
Esophagus - Gastroesophageal Junction
Esophagus - Mucosa
Esophagus - Muscularis
Fallopian Tube
Heart - Atrial Appendage
Heart - Left Ventricle
Kidney - Cortex
Liver
Lung
Minor Salivary Gland
Muscle - Skeletal
Nerve - Tibial
Ovary
Pancreas
Pituitary
Prostate
Skin - Not Sun Exposed (Suprapubic)
Skin - Sun Exposed (Lower leg)
Small Intestine - Terminal Ileum
Spleen
Stomach
Testis
Thyroid
Uterus
Vagina
Whole Blood
Tissues

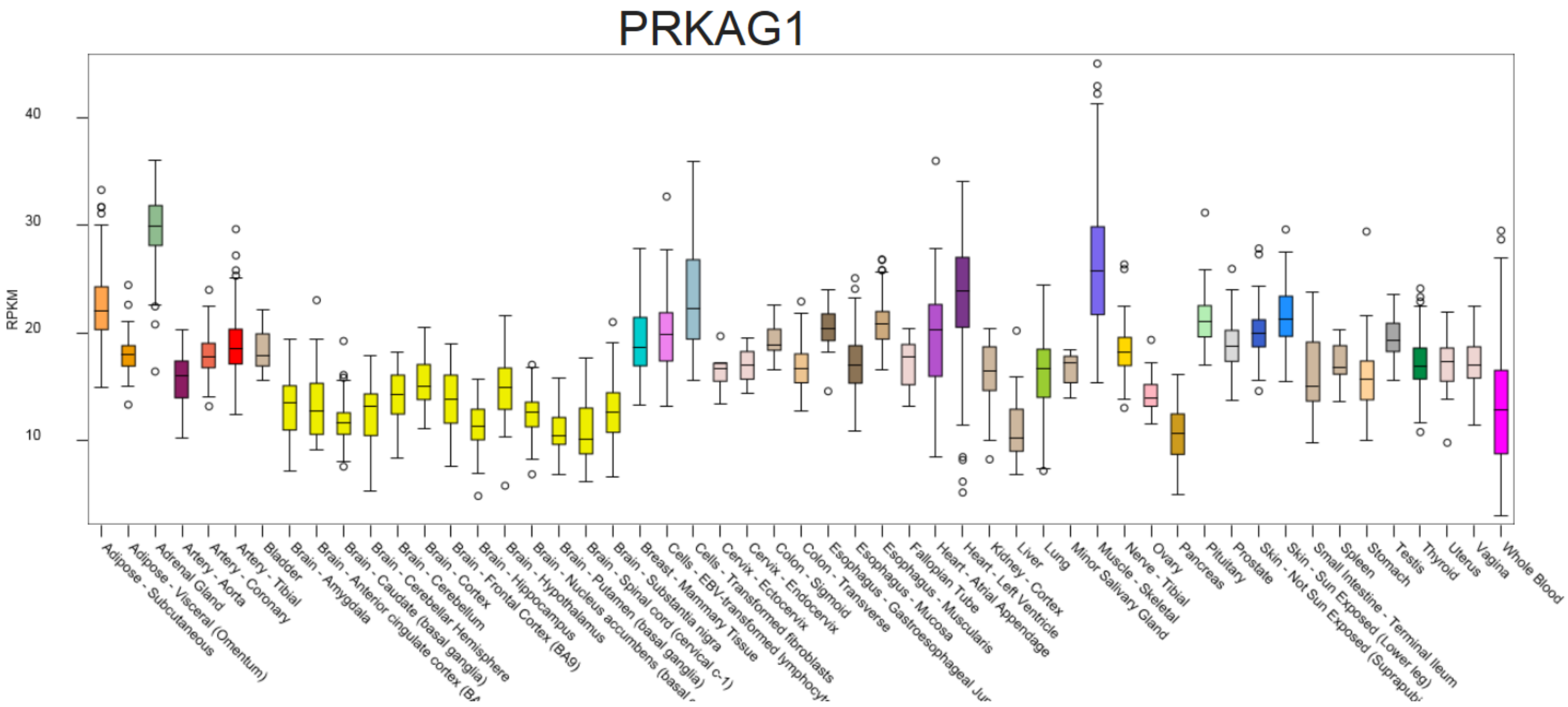

PRKAG1
RPKM
40
30
20
10
Adipose - Subcutaneous
Adipose - Visceral (Omentum)
Adrenal Gland
Artery - Aorta
Artery - Coronary
Artery - Tibial
Bladder
Brain - Amygdala
Brain - Anterior cingulate cortex (BA
Brain - Caudate (basal ganglia)
Brain - Cerebellar Hemisphere
Brain - Cerebellum
Brain - Cortex
Brain - Frontal Cortex (BA9)
Brain - Hippocampus
Brain - Hypothalamus
Brain - Nucleus accumbens (basal
Brain - Putamen (basal ganglia)
Brain - Spinal cord (cervical c-1)
Brain - Substantia nigra
Breast - Mammary Tissue
Cells - EBV-transformed lymphocy
Cells - Transformed fibroblasts
Cervix - Ectocervix
Cervix - Endocervix
Colon - Sigmoid
Colon - Transverse
Esophagus - Gastroesophageal Jun
Esophagus - Mucosa
Esophagus - Muscularis
Fallopian Tube
Heart - Atrial Appendage
Heart - Left Ventricle
Kidney - Cortex
Liver
Lung
Minor Salivary Gland
Muscle - Skeletal
Nerve - Tibial
Ovary
Pancreas
Pituitary
Prostate
Skin - Not Sun Exposed (Suprapub
Skin - Sun Exposed (Lower leg)
Small Intestine - Terminal Ileum
Spleen
Stomach
Testis
Thyroid
Uterus
Vagina
Whole Blood

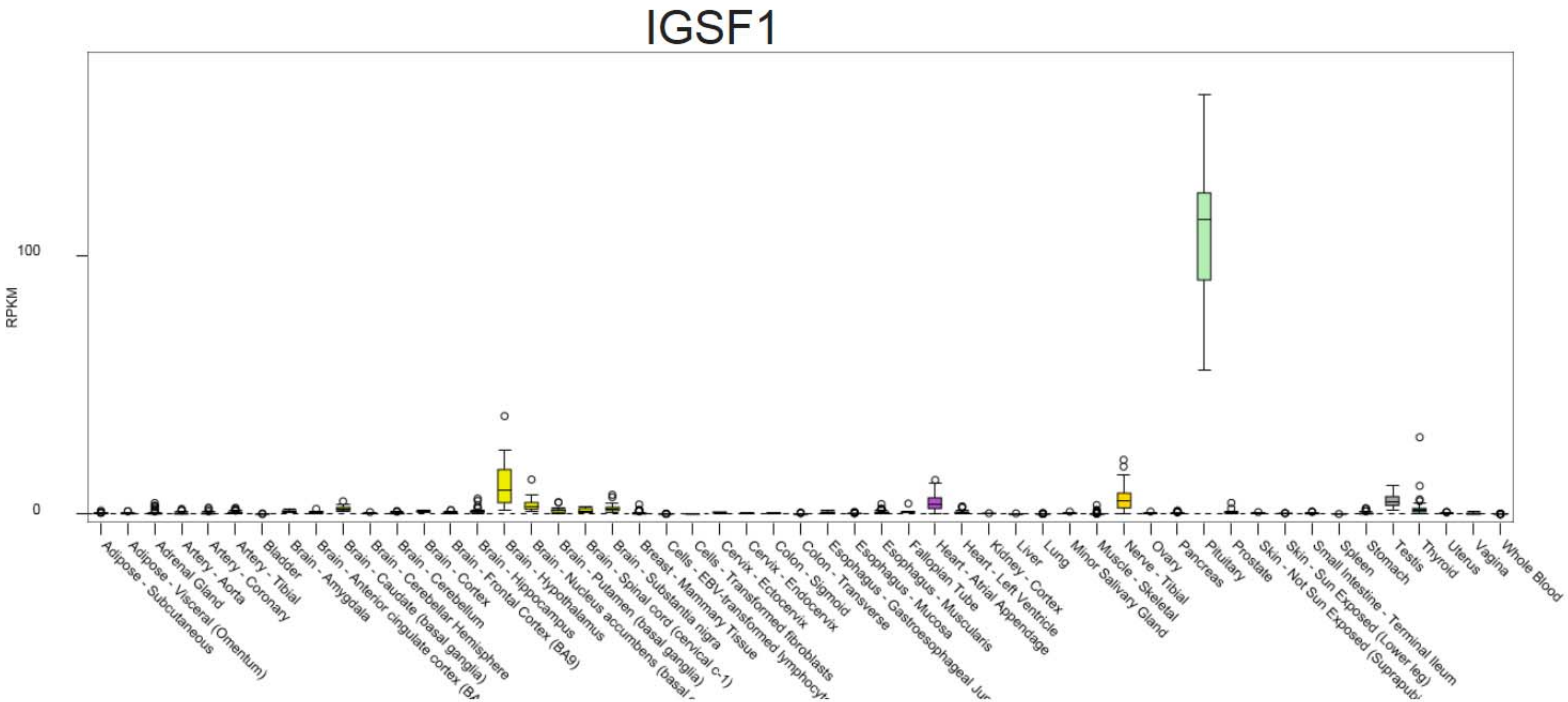
IGSF1
RPKM
100
0
Adipose - Subcutaneous
Adipose - Visceral (Omentum)
Adrenal Gland
Artery - Aorta
Artery - Coronary
Artery - Tibial
Bladder
Brain - Amygdala
Brain - Anterior cingulate cortex (BA
Brain - Caudate (basal ganglia)
Brain - Cerebellar Hemisphere
Brain - Cerebellum
Brain - Cortex
Brain - Frontal Cortex (BA9)
Brain - Hippocampus
Brain - Hypothalamus
Brain - Nucleus accumbens (basal
Brain - Putamen (basal ganglia)
Brain - Spinal cord (cervical c-1)
Brain - Substantia nigra
Breast - Mammary Tissue
Cells - EBV-transformed lymphocyte
Cells - Transformed fibroblasts
Cervix - Ectocervix
Cervix - Endocervix
Colon - Sigmoid
Colon - Transverse
Esophagus - Gastroesophageal Ju
Esophagus - Mucosa
Esophagus - Muscularis
Fallopian Tube
Heart - Atrial Appendage
Heart - Left Ventricle
Kidney - Cortex
Liver
Lung
Minor Salivary Gland
Muscle - Skeletal
Nerve - Tibial
Ovary
Pancreas
Pituitary
Prostate
Skin - Not Sun Exposed (Suprapub
Skin - Sun Exposed (Lower leg)
Small Intestine - Terminal Ileum
Spleen
Stomach
Testis
Thyroid
Uterus
Vagina
Whole Blood

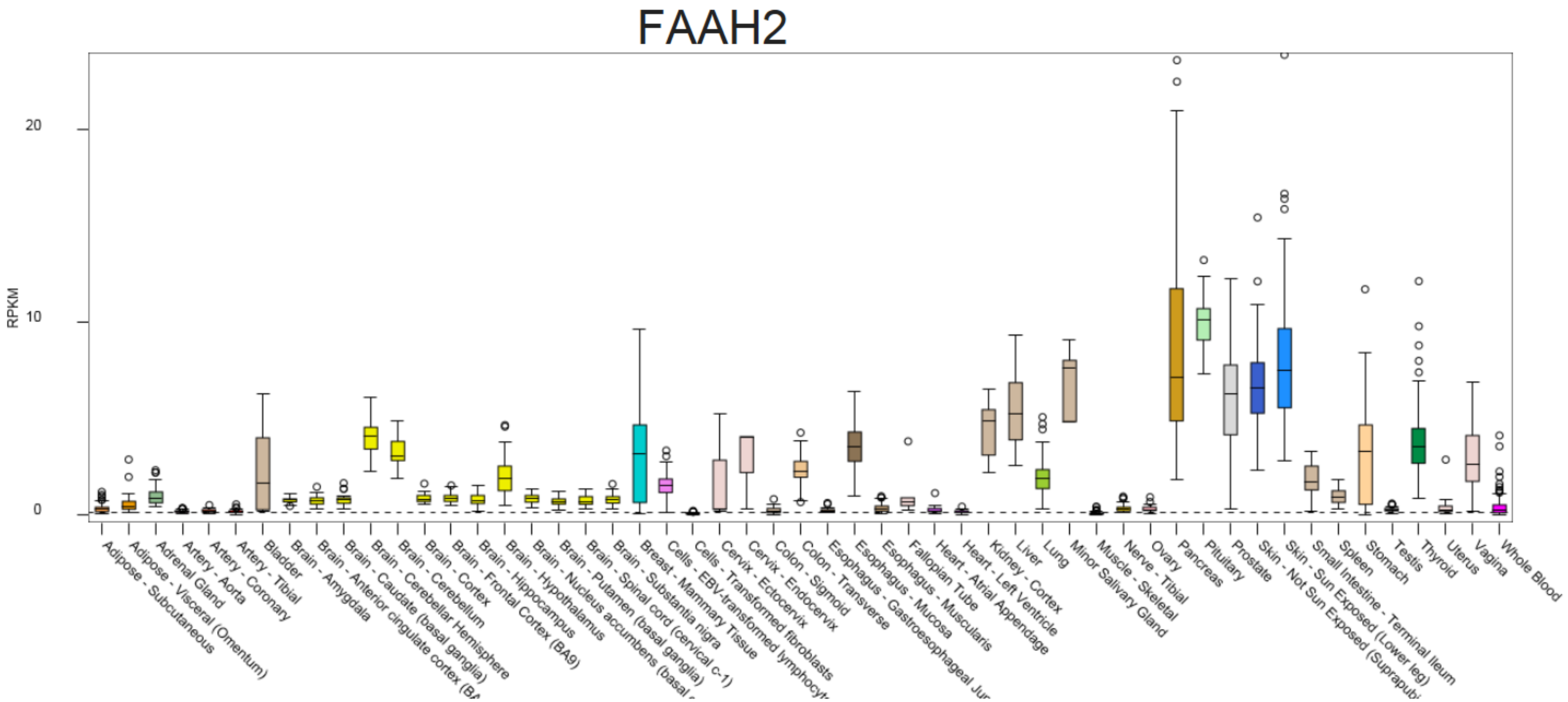
FAAH2
RPKM
0
10
20
Adipose - Subcutaneous
Adipose - Visceral (Omentum)
Adrenal Gland
Artery - Aorta
Artery - Coronary
Artery - Tibial
Bladder
Brain - Amygdala
Brain - Anterior cingulate cortex (B
Brain - Caudate (basal ganglia)
Brain - Cerebellar Hemisphere
Brain - Cerebellum
Brain - Cortex
Brain - Frontal Cortex (BA9)
Brain - Hippocampus
Brain - Hypothalamus
Brain - Nucleus accumbens (basal
Brain - Putamen (basal ganglia)
Brain - Spinal cord (cervical c-1)
Brain - Substantia nigra
Breast - Mammary Tissue
Cells - EBV-transformed lymphocyt
Cells - Transformed fibroblasts
Cervix - Ectocervix
Cervix - Endocervix
Colon - Sigmoid
Colon - Transverse
Esophagus - Gastroesophageal Jun
Esophagus - Mucosa
Esophagus - Muscularis
Fallopian Tube
Heart - Atrial Appendage
Heart - Left Ventricle
Kidney - Cortex
Liver
Lung
Minor Salivary Gland
Muscle - Skeletal
Nerve - Tibial
Ovary
Pancreas
Pituitary
Prostate
Skin - Not Sun Exposed (Suprapubi
Skin - Sun Exposed (Lower leg)
Small Intestine - Terminal Ileum
Spleen
Stomach
Testis
Thyroid
Uterus
Vagina
Whole Blood

Supplementary Table 1 | Descriptive summary statistics for all included GWAS studies

Type	Study Name / acronym	Full Study name	N	Mean age (SD)	Mean AAM (SD)	Mean birth year	Mean BMI (SD)
Discovery - European	1958BC	1958 National Child Development Study (also known as the 1958 Birth Cohort Study)	1,836	16	12.83 (1.26)	1958	NA (used BMI z-score at closest age to menarche)
Discovery - European	ARIC	Atherosclerosis Risk in Communities Study	3,779	NA	12.93 (1.52)	1932	26.6 (5.4)
Discovery - European	Fenland		611	NA	12.8 (1.5)	1961	26.6 (5.3)
Discovery - European	FHS	Framingham Heart Study	3,673	42.5 (10.1)	12.8 (1.5)	1952	25.3 (5.6)
Discovery - European	INGI-VB		933	54.7 (17.9)	12.9 (1.5)	1950	25.6(4.9)
Discovery - European	InterAct Cases	The EPIC-InterAct Study	1,276	55.9 (7.9)	13.0 (1.7)	1939	30.3 (5.4)
Discovery - European	InterAct Sub-cohort	The EPIC-InterAct Study	2,916	52.0 (9.5)	13.1 (1.5)	1943	25.6 (4.4)
Discovery - European	KORA F4	Cooperative Health Research in the Region of Augsburg (follow-up 4)	1,439	55.26 (13.07)	13.46 (1.49)	1951	27.24 (5.29)
Discovery - European	Rotterdam	Rotterdam Study	1,564	70.9 (9.0)	13.5 (1.6)	1920	26.7 (4.1)
Discovery - European	SHIP	Study of Health in Pomerania (follow-up 2)	1,136	56.04 (13.06)	13.49 (1.55)	1953	26.32 (5.00)
Discovery - European	SHIP-TREND	Study of Health in Pomerania - TREND	1,718	51.03 (14.85)	13.31 (1.52)	1958	27.58 (5.58)
Discovery - European	WGHS	Women's Genome Health Study	22,199	54.2 (7.1)	12.4 (1.4)	1939	25.9 (5.0)
Discovery - European	WHI	Women's Health Initiative	17,962	66.3	NA	NA	28.3
Discovery - European	Cambridge Cancer	The EMBRACE, SEARCH (breast cancer and ovarian cancer) and SIBS studies	5,575	54.3 (9.06)	12.8 (1.53)	1948	22.8 (4.38)
Discovery - European	Amish	Old Order Amish Study	828	49.1 (3.7)	13.1 (1.3)	1953	28.4 (5.7)
Discovery - European	EGCUT	Estonian Genome Center, University of Tartu	2,295	48.64 (17.12)	13,42 (1,47)	1958	28.56 (8.65)
Discovery - European	Sardinia	SardiNIA	3,661	43.27 (17.31)	13.12 (1.56)	1960	24.69 (4.99)
Discovery - European	Korcula	CROATIA Korcula	453	54.86 (14.09)	13.7 (1.63)	1952	27.47 (4.14)
Discovery - European	RAINE	Western Australian Pregnancy Cohort (Raine) Study	532	14.07 (0.185)	12.776 (NA)	1989	21.99 (4.039)
Discovery - European	Generation Scotland	Generation Scotland:Scottish Family Health Study	2,271	51.6 (13.64)	12.85 (1.47)	1956	26.7 (5.19)
Replication - European	23andMe	The 23andMe Research Program	76,831	NA	NA	NA	NA
Replication - European	deCODe	deCODE Genetics, Iceland	39,486	NA	13.1 (1.3)	1948	NA

Supplementary Table 2 | – Exome array genotyping information by study

Study	Exome chip version	CHARGE common calling?	Called using CHARGE cluster file?	Called using CHARGE "best practices" protocol?	Additional QC info
1958BC	HumanExome BeadChip-12v1_A	N	N	N	Genotyped as part of UK Exome Chip Consortium. QC according to EXOME-CHIP QUALITY CONTROL SOP Version 5, 2012-11-20 (SOP v5)
ARIC	HumanExome BeadChip v1.0	Y	N/A	Y	-
Fenland	HumanExome BeadChip v1.0	N	N, Fenland + EPIC called together, (2042 individuals)	N	Gencall + zcall
FHS	HumanExome BeadChip v1.0	Y	N/A	Y	-
INGI-VB	HumanExome-12v1-2_A, HumanOmniExpressExome-8v1-2	N	N, 1787 individuals used in calling	N	Zcall genotyping. Variants were coded as the minor allele from the CHARGE Joint calling
InterAct Cases+ sub-cohort	Illumina HumanCoreExome chip	N	N, All InterAct called together (7397 individuals)	N	Gencall + zcall
KORA F4	HumanExome BeadChip v1.0	N	Y	Y	-
Rotterdam	HumanExome BeadChip v1.0	Y	N/A	Y	-
SHIP	HumanExome BeadChip v1.0	N	N, included in 7366 individuals called together	N	Gencall + SOPv5 (PMID: 24777453). Samples called and analysed from these two studies jointly.
SHIP-TREND	HumanExome BeadChip v1.0	N		N	
WGHS	HumanExome BeadChip v1.1A	N	N. 22,618 individuals used in calling	Y	-
WHI	HumanExome BeadChip v1.0 (n ~ 23,000)and v1.1 (n~1000)	N	N, called within substudy	N	GenomeStudio v2010.3; QC described in Auer et al. Rare and low-frequency coding variants in CXCR2 and other genes are associated with hematological traits. Nature genetics 46, 629-634 (2014).
Cambridge Cancer	HumanExome BeadChip v1.0	N	Y	Y	-
Amish	HumanExome BeadChip v1.0	N	N. 1909 individuals used in calling	Y	AdditionalQC was to compare overlapping genotypes with previously run chips to check concordance rates
EGCUT	HumanExome-12v1-1	N	N, 1927 and 2894 called in separate batches	N	Called with GENCALL and subsequently zCALL, with quality control performed as described in Exome Chip Quality Control SOP Version 5, 2012-11-20
Sardinia	HumanExome BeadChip-12v1_A	N	N, 6713 individuals used in calling	N	-
Korcula	HumanExome BeadChip-12v1_A	N	Y	Y	Some additional manual checking and reclustering
RAINE	HumanExome BeadChip-12v1_A	N	N	N	GenomeStudio for calling
Generation Scotland	HumanExome BeadChip-12v1_A	N	Y	Y	Some additional manual checking and reclustering

Supplementary Table 3 - Contributing study acknowledgements

Study Name	Acknowledgements	Funding	Disclosure
1958 Birth Cohort	This work made use of data and samples generated by the 1958 Birth Cohort (NCDS). Access to these resources was enabled via the 58READIE Project funded by Wellcome Trust and Medical Research Council (grant numbers WT095219MA and G1001799). A full list of the financial, institutional and personal contributions to the development of the 1958 Birth Cohort Biomedical resource is available at http://www2.le.ac.uk/projects/birthcohort. Genotyping was undertaken as part of the Wellcome Trust Case-Control Consortium (WTCCC) under Wellcome Trust award 076113, and a full list of the investigators who contributed to the generation of the data is available at www.wtccc.org.uk.	Wellcome Trust grant WT095219MA, Medical Research Council grant G1001799, Wellcome Trust award 076113.	None
ARIC	The Atherosclerosis Risk in Communities (ARIC) study is carried out as a collaborative study supported by the National Heart, Lung, and Blood Institute (NHLBI) contracts (HHSN268201100005C, HHSN268201100006C, HHSN268201100007C, HHSN268201100008C, HHSN268201100009C, HHSN268201100010C, HHSN268201100011C, and HHSN268201100012C). The authors thank the staff and participants of the ARIC study for their important contributions. Funding support for "Building on GWAS for NHLBI-diseases: the U.S. CHARGE consortium" was provided by the NIH through the American Recovery and Reinvestment Act of 2009 (ARRA) (5RC2HL102419).	R21HL123677-01, 1R01HL118305-01A1(NF)	None
WHI	The WHI program is funded by the National Heart, Lung, and Blood Institute, National Institutes of Health, U.S. Department of Health and Human Services through contracts HHSN268201100046C, HHSN268201100001C, HHSN268201100002C, HHSN268201100003C, HHSN268201100004C, and HHSN271201100004C.	R21HL123677-01, 1R01HL118305-01A1(NF)	None
InterAct	We thank all EPIC participants and staff for their contribution to the study. We thank staff from the Technical, Field Epidemiology and Data Functional Group Teams of the MRC Epidemiology Unit in Cambridge, UK, for carrying out sample preparation, DNA provision and quality control, genotyping and data-handling work.	The EPIC-InterAct study received funding from the European Union (Integrated Project LSHM-CT-2006-037197 in the Framework Programme 6 of the European Community).	None

Study Name	Acknowledgements	Funding	Disclosure
FHS	The authors thank the Framingham Heart Study participants and staff.	The Framingham Heart Study phenotype-genotype analyses were supported by the National Institute of Aging (Genetics of Reproductive Life Period and Health Outcomes, R21AG032598; JMM, KL and R01AG29451 JMM, KL). The Framingham Heart Study of the National Heart Lung and Blood Institute of the National Institutes of Health and Boston University School of Medicine was supported by the National Heart, Lung and Blood Institute's Framingham Heart Study Contract No. N01-HC-25195 and its contract with Affymetrix, Inc for genotyping services (Contract No. N02-HL-6-4278). Analyses reflect intellectual input and resource development from the Framingham Heart Study investigators participating in the SNP Health Association Resource (SHARe) project. A portion of this research was conducted using the Linux Cluster for Genetic Analysis (LinGA-II) funded by the Robert Dawson Evans Endowment of the Department of Medicine at Boston University School of Medicine and Boston Medical Center. Genotyping, quality control and calling of the Illumina HumanExome BeadChip in the Framingham Heart Study was supported by funding from the National Heart, Lung and Blood Institute Division of Intramural Research (Daniel Levy and Christopher J. O'Donnell, Principal Investigators).	None
KORA F4	We thank all the study participants, all members of staff of the Institutes of Epidemiology and the field staff in Augsburg who planned and conducted the study.	The KORA study group consists of A. Peters (speaker), R. Holle, K. Strauch, J. Heinrich, R. Leidl, C. Meisinger, and their co-workers, who are responsible for the design and conduct of the KORA studies.<br>The KORA research platform (KORA, Cooperative Health Research in the Region of Augsburg) was initiated and financed by the Helmholtz Zentrum München - German Research Center for Environmental Health, which is funded by the German Federal Ministry of Education and Research and by the State of Bavaria. Furthermore, KORA research was supported within the Munich Center of Health Sciences (MC Health), Ludwig-Maximilians-Universität, as part of LMUinnovativ. The funders had no role in study design, data collection and analysis, decision to publish, or preparation of the manuscript.<br>Elisabeth Altmaier - European Union's Seventh Framework Programme (FP7-Health-F5-2012) under Grant agreement No 305280 (MIMOmics).	No conflict of interest
Women's Genome Health Study (WGHS)		The WGHS is supported by HL043851 and HL080467 from the National Heart, Lung, and Blood Institute and CA047988 from the National Cancer Institute, and the Donald W. Reynolds Foundation, with collaborative scientific support and funding for genotyping provided by Amgen.	Support from Amgen to PMR and DIC for the WGHS

Name	Acknowledgements	Funding	Disclosure
SHIP/SHIP-TREND	SHIP is part of the Community Medicine Research net of the University of Greifswald, Germany, which is funded by the Federal Ministry of Education and Research (grants no. 01ZZ9603, 01ZZ0103, and 01ZZ0403), the Ministry of Cultural Affairs as well as the Social Ministry of the Federal State of Mecklenburg-West Pomerania, and the network 'Greifswald Approach to Individualized Medicine (GANI_MED)' funded by the Federal Ministry of Education and Research (grant 03IS2061A). ExomeChip data have been supported by the Federal Ministry of Education and Research (grant no. 03Z1CN22). The University of Greifswald is a member of the Caché Campus program of the InterSystems GmbH. The generation of genome-wide data was supported by funds of Siemens Healthcare and the Federal State of Mecklenburg-West Pomerania.	Grants no. 01ZZ9603, 01ZZ0103, 01ZZ0403, 03Z1CN22 and 03IS2061A	None
Fenland	The Fenland Study is funded by the Wellcome Trust and the Medical Research Council, as well as by the Support for Science Funding programme and CamStrad. We are grateful to all the volunteers for their time and help, and to the General Practitioners and practice staff for help with recruitment. We thank the Fenland Study co-ordination team and the Field Epidemiology team of the MRC Epidemiology Unit for recruitment and clinical testing.		None
CROATIA Korcula	Exome array genotyping was performed at the Wellcome Trust Clinical Research Facility Genetics Core at Western General Hospital, Edinburgh, UK. We would like to acknowledge the invaluable contributions of the recruitment team in Korcula, the administrative teams in Croatia and Edinburgh and the people of Korcula.	Medical Research Council UK, the Ministry of Science, Education and Sport in the Republic of Croatia (number 216-10803150302) and the Croatian Science Foundation (grant 8875).	None
Amish	None	U01-HL72515, U01-HL84756, R01-088119, P30-DK072488, K01-HL116770	None
Raine	This study was supported by the National Health and Medical Research Council of Australia [grant numbers 403981 and 003209] and the Canadian Institutes of Health Research [grant number MOP-82893]. The authors are grateful to the Raine Study participants and their families, and to the Raine Study research staff for cohort coordination and data collection. The authors gratefully acknowledge the NH&MRC for their long term contribution to funding the study over the last 20 years and also the following Institutions for providing funding for Core Management of the Raine Study: The University of Western Australia (UWA), Raine Medical Research Foundation, UWA Faculty of Medicine, Dentistry and Health Sciences, The Telethon Institute for Child Health Research and Women and Infants Research Foundation. The authors gratefully acknowledge the assistance of the Western Australian DNA Bank (National Health and Medical Research Council of Australia National Enabling Facility).		None

Name	Acknowledgements	Funding	Disclosure
EMBRACE/SIBS/ SEARCH Exome Chip genotyping,<br><br>SIBS<br><br>SEARCH<br><br>EMBRACE	Douglas F. Easton is the PI of the study. EMBRACE Collaborating Centres are:<br>Coordinating Centre, Cambridge:Debra Frost, Steve Ellis, Radka Platte, Jo Perkins.<br>North of Scotland Regional Genetics Service, Aberdeen: Zosia Miedzybrodzka, Helen Gregory.<br>Northern Ireland Regional Genetics Service, Belfast: Patrick Morrison, Lisa Jeffers.<br>West Midlands Regional Clinical Genetics Service, Birmingham: Kai-ren Ong, Jonathan Hoffman.<br>South West Regional Genetics Service, Bristol: Alan Donaldson, Margaret James.<br>East Anglian Regional Genetics Service, Cambridge: Joan Paterson, Marc Tischkowitz, Sarah Downing, Amy Taylor.<br>South West Regional Genetics Service, Bristol: Alan Donaldson, Margaret James.<br>East Anglian Regional Genetics Service, Cambridge: Joan Paterson, Marc Tischkowitz, Sarah Downing, Amy Taylor.<br>Medical Genetics Services for Wales, Cardiff: Alexandra Murray, Mark T. Rogers, Emma McCann.<br>St James's Hospital, Dublin & National Centre for Medical Genetics, Dublin: M. John Kennedy, David Barton.<br>Peninsula Clinical Genetics Service, Exeter: Carole Brewer, Emma Kivuva, Anne Searle, Selina Goodman, Kathryn Hill.<br>West of Scotland Regional Genetics Service, Glasgow: Rosemarie Davidson, Victoria Murday, Nicola Bradshaw, Lesley Snadden, Mark Longmuir, Catherine Watt, Sarah Gibson, Eshika Haque, Ed Tobias, Alexis Duncan.<br>South East Thames Regional Genetics Service, Guy's Hospital London: Louise Izatt, Chris Jacobs, Caroline Langman.<br>North West Thames Regional Genetics Service, Harrow: Huw Dorkins.<br>Leicestershire Clinical Genetics Service, Leicester: Julian Barwell<br>Yorkshire Regional Genetics Service, Leeds: Julian Adlard, Gemma Serra-Feliu.<br>Cheshire & Merseyside Clinical Genetics Service, Liverpool: Ian Ellis, Claire Foo.<br>Manchester Regional Genetics Service, Manchester: D Gareth Evans, Fiona Lalloo, Jane Taylor.<br>North East Thames Regional Genetics Service, NE Thames, London: Lucy Side, Alison Male, Cheryl Berlin.<br>Nottingham Centre for Medical Genetics, Nottingham: Jacqueline Eason, Rebecca Collier.<br>Northern Clinical Genetics Service, Newcastle: Alex Henderson, Oonagh Claber, Irene Jobson.<br>Oxford Regional Genetics Service, Oxford: Lisa Walker, Diane McLeod, Dorothy Halliday, Sarah Durell, Barbara Stayner.<br>The Institute of Cancer Research and Royal Marsden NHS Foundation Trust: Ros Eeles, Nazneen Rahman, Elizabeth Bancroft, Elizabeth Page, Audrey Ardern-Jones, Kelly Kohut, Jennifer Wiggins, Jenny Pope, Sibel Saya, Natalie Taylor, Zoe Kemp and Angela George<br>North Trent Clinical Genetics Service, Sheffield: Jackie Cook, Oliver Quarrell, Cathryn Bardsley.<br>South West Thames Regional Genetics Service, London: Shirley Hodgson, Sheila Goff, Glen Brice, Lizzie Winchester, Charlotte Eddy, Vishakha Tripathi, Virginia Attard.<br>Wessex Clinical Genetics Service, Princess Anne Hospital, Southampton: Diana Eccles, Anneke Lucassen, Gillian Crawford, Donna McBride, Sarah Smalley.	SIBS - CRUK ref: C1287/A8459<br>SEARCH - CRUK ref: A490/A10124<br>EMBRACE is supported by Cancer Research UK Grants C1287/A10118, C1287/A16563 and C1287/A17523. Genotyping was supported by Cancer Research–UK grant C12292/A11174D and C8197/A16565. Gareth Evans and Fiona Lalloo are supported by an NIHR grant to the Biomedical Research Centre, Manchester. The Investigators at The Institute of Cancer Research and The Royal Marsden NHS Foundation Trust are supported by an NIHR grant to the Biomedical Research Centre at The Institute of Cancer Research and The Royal Marsden NHS Foundation Trust. Ros Eeles and Elizabeth Bancroft are supported by Cancer Research UK Grant C5047/A8385.	None

Name	Acknowledgements	Funding	Disclosure
INGI-VB	We thank the inhabitants of the VB that made this study possible, the local administrations, the Tortona and Genova archdiocese and the ASL-22, Novi Ligure (Al) for support. We also thank Clara Camaschella for data collection supervision and organization of the clinical data collection, Fiammetta Viganò for technical help, Corrado Masciullo and Massimiliano Cocca for building the analysis platform.	The research was supported by funds from Fondazione Cariplo, Italy, Ministry of Health, Ricerca Finalizzata 2011-2012, Ministry of Health CCM 2010 and Telethon, Italy to DT	The funders had no role in study design, data collection and analysis, decision to publish, or preparation of the manuscript.
SardiNIA	We thank all the volunteers and all the staff for their contribution to the study.	This study was funded in part by the National Institutes of Health (National Institute on Aging, National Heart Lung and Blood Institute, and National Human Genome Research Institute). This research was supported by National Human Genome Research Institute grants HG005581, HG005552, HG006513, HG007089, HG007022, and HG007089; by National Heart Lung and Blood Institute grant HL117626; by the Intramural Research Program of the NIH, National Institute on Aging, with contracts N01-AG-1-2109 and HHSN271201100005C; by Sardinian Autonomous Region (L.R. no. 7/2009) grant cRP3-154; by grant FaReBio2011 "Farmaci e Reti Biotecnologiche di Qualità".	None
Generation Scotland	Exome array genotyping was performed at the Wellcome Trust Clinical Research Facility Genetics Core at Western General Hospital, Edinburgh, UK. We would like to acknowledge the invaluable contributions of the families who took part in the Generation Scotland: Scottish Family Health Study, the general practitioners and Scottish School of Primary Care for their help in recruiting them, and the whole Generation Scotland team, which includes academic researchers, IT staff, laboratory technicians, statisticians and research managers.	Scottish Executive Health Department, Chief Scientist Office, grant number CZD/16/6. Exome array genotyping for GS:SFHS was funded by the Medical Research Council UK	None
23andMe	We would like to thank the customers and employees of 23andMe for making this work possible.	This work was supported in part by NIH Award 2R44HG006981-02 from the National Human Genome Research Institute.	None

Name	Acknowledgements	Funding	Disclosure
Rotterdam	The generation and management of the Illumina exome chip v1.0 array data for the Rotterdam Study (RS-I) was executed by the Human Genotyping Facility of the Genetic Laboratory of the Department of Internal Medicine, Erasmus MC, Rotterdam, The Netherlands. The Exome chip array data set was funded by the Genetic Laboratory of the Department of Internal Medicine, Erasmus MC, from the Netherlands Genomics Initiative (NGI)/Netherlands Organisation for Scientific Research (NWO)-sponsored Netherlands Consortium for Healthy Aging (NCHA; project nr. 050-060-810); the Netherlands Organization for Scientific Research (NWO; project number 184021007) and by the Rainbow Project (RP10; Neterlands Exome Chip Project) of the Biobanking and Biomolecular Research Infrastructure Netherlands (BBMRI-NL; www.bbmri.nl). We thank Ms. Mila Jhamai, Ms. Sarah Higgins, and Mr. Marijn Verkerk for their help in creating the exome chip database, and Carolina Medina-Gomez, BSc, Lennard Karsten, BSc, and Dr. Linda Broer for QC, variant calling and providing data descriptives. Variants were called using the best practice protocol developed by Grove et al. as part of the CHARGE consortium exome chip central calling effort (Grove et al., PLoS One, 2014).<br><br>The Rotterdam Study is funded by Erasmus Medical Center and Erasmus University, Rotterdam, Netherlands Organization for the Health Research and Development (ZonMw), the Research Institute for Diseases in the Elderly (RIDE), the Ministry of Education, Culture and Science, the Ministry for Health, Welfare and Sports, the European Commission (DG XII), and the Municipality of Rotterdam. The authors are grateful to the study participants, the staff from the Rotterdam Study and the participating general practitioners and pharmacists.		None
EGCUT		EGCUT work was supported by the Estonian Ministry of Science and Education grant IUT20-60; the US National Institute of Health [R01DK075787]; the Development Fund of the University of Tartu (grant SP1GVARENG); the European Regional Development Fund to the Centre of Excellence in Genomics (EXCEGEN; grant 3.2.0304.11-0312); and through FP7 grant 313010.	None
